# Intranasal Delivery of miR-146a Mimics Delayed Seizure Onset in the Lithium-Pilocarpine Mouse Model

**DOI:** 10.1155/2017/6512620

**Published:** 2017-01-24

**Authors:** Hua Tao, Jianghao Zhao, Tingting Liu, Yujie Cai, Xu Zhou, Huaijie Xing, Yan Wang, Mingkang Yin, Wangtao Zhong, Zhou Liu, Keshen Li, Bin Zhao, Haihong Zhou, Lili Cui

**Affiliations:** ^1^Guangdong Key Laboratory of Age-Related Cardiac and Cerebral Diseases, Institute of Neurology, Affiliated Hospital of Guangdong Medical University, Zhanjiang, Guangdong, China; ^2^Clinical Research Center, Affiliated Hospital of Guangdong Medical University, Zhanjiang, Guangdong, China

## Abstract

Unveiling the key mechanism of temporal lobe epilepsy (TLE) for the development of novel treatments is of increasing interest, and anti-inflammatory miR-146a is now considered a promising molecular target for TLE. In the current study, a C57BL/6 TLE mouse model was established using the lithium-pilocarpine protocol. The seizure degree was evaluated according to the Racine scale, and level 5 was considered the threshold for generalized convulsions. Animals were sacrificed to analyze the hippocampus at three time points (2 h and 4 and 8 weeks after pilocarpine administration to evaluate the acute, latent, and chronic phases, resp.). After intranasal delivery of miR-146a mimics (30 min before pilocarpine injection), the percent of animals with no induced seizures increased by 6.7%, the latency to generalized convulsions was extended, and seizure severity was reduced. Additionally, hippocampal damage was alleviated. While the relative miR-146a levels significantly increased, the expression of its target mRNAs (IRAK-1 and TRAF-6) and typical inflammatory modulators (NF-*κ*B, TNF-*α*, IL-1*β*, and IL-6) decreased, supporting an anti-inflammatory role of miR-146a via the TLR pathway. This study is the first to demonstrate that intranasal delivery of miR-146a mimics can improve seizure onset and hippocampal damage in the acute phase of lithium-pilocarpine-induced seizures, which provides inflammation-based clues for the development of novel TLE treatments.

## 1. Introduction

Many antiepileptic drugs (AEDs) have been developed for seizure treatment in past decades, although almost one-third of epileptic patients are resistant to these AEDs [1–4]. Drug resistance likely originates from an undetermined pathogenesis of epilepsy. Strikingly, temporal lobe epilepsy (TLE), a subtype of epilepsy and the most common cause of partial seizures, represents the primary source of refractory epilepsy [5–7]. Hence, increasing interest has focused on unveiling the precise mechanism of TLE in order to develop novel therapies for seizure treatment.

Increasing clinical and experimental studies have indicated that neuroinflammation is one of the key mechanisms responsible for TLE. Inflammatory mediators, such as tumor necrosis factors alpha (TNF-*α*), interleukin 1 beta (IL-1*β*), interleukin 6 (IL-6), and toll-like receptor 4 (TLR4), have been reported to be abnormally expressed in the hippocampus of TLE rodents and TLE patients [8–10]. In addition, recurrent seizures can augment neuroinflammation by inducing the recruitment of peripheral inflammatory cells [10–12]. Lipopolysaccharide (LPS), a typical exogenous inducer of inflammation, was shown to lower the seizure threshold and increase discharges in rodents by triggering a cascade of inflammatory cytokines, such as TNF-*α* and IL-1*β* [13, 14]. Moreover, risk factors for TLE, such as febrile convulsions, are usually accompanied by inflammatory responses in the brain [15]. In a series of in vivo studies, anti-inflammatory treatments, such as aspirin, minocycline and rapamycin, significantly reduced epileptic activities by inhibiting cyclooxygenase, microglia activation, and LPS-dependent inflammation, respectively [14, 16, 17]. However, no anti-inflammatory AEDs have been successfully developed for TLE in clinical practice, likely because of the lack of a promising molecular target.

MicroRNAs (miRNAs) are small noncoding RNAs that negatively regulate the translation of their target messenger RNAs (mRNAs). Abnormal expression of miRNAs is implicated in almost all pathological processes, including but not limited to neuroinflammation and neurodegeneration [18–20]. In light of existing evidence, miR-146a represents one of the most important inflammatory miRNAs [21–24]. The expression of miR-146a is strongly induced by IL-1*β* and inhibited by the IL-1*β* receptor antagonist in astrocytes, and modulation of miR-146a expression was shown to negatively regulate the expression of downstream molecules, including inflammation-related interleukin-1 receptor-associated kinase 1 (IRAK-1) and TNF receptor-associated factor 6 (TRAF-6), as well as the release of TNF-*α* and IL-6 [25]. Moreover, the negative relationship between IL-1*β* and miR-146a has been demonstrated in the hippocampi of an immature rat model and patients with TLE [8]. Our recent study also reported that a functional polymorphism of the miR-146a gene was associated with susceptibility to drug-resistant epilepsy, which included primarily TLE cases [26]. Thus, we believe that miR-146a may represent a promising molecular target for TLE. In this study, the lithium-pilocarpine model was established to evaluate whether intranasal delivery of miR-146a mimics could delay seizure onset in TLE.

## 2. Materials and Methods

### 2.1. Experimental Animals

This study included a total of 250 C57BL/6 7-week-old postnatal mice obtained from the Animal Center of Guangdong Medical University, Zhanjiang, China. All of these mice were bred under a controlled temperature of 22°C to 26°C and a humidity of 55% to 65%. A light-dark cycle was implemented in accordance with natural day and night alterations. After a one-week adaptation to the environment with free access to food and water, the mice (8-week postnatal, 25.14 ± 1.78 g) were used in the experiments. The study protocol was conducted according to the Guide for the Care and Use of Laboratory Animals (Ministry of Science and Technology of China, 2006) and was approved by the Animal Ethics Committee of Guangdong Medical University.

### 2.2. Dynamic Changes in Relative miR-146a Levels after Intranasal Delivery of miR-146a Mimics

Following the manufacturer's instructions, 20 nmol of miR-146a mimics (Catalog number: M02010; Genesky Biotech, Shanghai, China) was dissolved in 1 mL RNase-free water and then stored at −20°C before intranasal administration for the subsequent experiments. Referring to the previous study [27], mice were fixed using a simple mouse holder (Yuyan Instruments, Shanghai, China) and placed in a supine position; the miR-146a mimic solution (20 nmol/mL, 1 mL) was then administered via a pipette in 1 *μ*L drops, alternating between each naris every 2-3 min. A total of thirty mice were randomly selected and equally divided into three groups to receive miR-146a mimics (20 nmol/mL, 0.25 mL/kg body weight). The three groups were sacrificed individually for collection of the hippocampus at 15 min, 30 min, and 1 h after intranasal delivery. Another group of ten mice received RNase-free water (0.25 mL/kg body weight) as control and were sacrificed immediately after vehicle administration (0 min). Finally, the relative miR-146a levels in the hippocampus were quantitated using real-time quantitative polymerase chain reaction (qPCR), and the dynamic changes in the relative miR-146a levels after intranasal delivery of the miR-146a mimics were further analyzed and compared with the vehicle control.

### 2.3. Epileptiform Discharges in the Lithium-Pilocarpine Model

To confirm epileptiform discharges in the lithium-pilocarpine model, a tethered electroencephalogram/electromyography (EEG/EMG) acquisition system (Pinnacle Technologies Inc., Lawrence, USA) was applied in the present study. Here, three mice were randomly selected and anesthetized using 3% chloral hydrate (10 mL/kg body weight, intraperitoneally (i.p.), Sigma-Aldrich, St. Louis, USA). Once awareness was deprived, four pilot holes were drilled through the skull for the implantation of intracranial electrodes, and a preamplifier head mount was affixed to the skull using dental acrylic, as described in a previous study [28]. After a one-week recovery period with free access to food and water, no abnormalities, such as infection, hemorrhage, or seizure, were observed. Subsequently, the mice underwent the lithium-pilocarpine protocol as follows. Lithium chloride (125 mg/kg body weight, i.p.; Amresco LLC, Solon, China) was used to enhance the sensitivity of pilocarpine, scopolamine (1 mg/kg body weight, i.p.; Tomax, Shenzhen, China) was injected 20 h later to limit cholinergic effects and the risk of unexpected death, and pilocarpine (200 mg/kg body weight, i.p.; Cayman, Ann Arbor, USA) was administered 30 min later. The degree of seizures was evaluated according to the Racine scale, and level 5 was considered as the threshold for generalized convulsions [29, 30]. Epileptiform discharges were recorded within 1 h after the administration of pilocarpine.

### 2.4. Grouping and Experimental Procedures

The C57BL/6 8-week-old postnatal mice were randomly divided into three groups: the experimental group, which was used for the induction of the lithium-pilocarpine model (*n* = 69), the treatment group (*n* = 69), and the vehicle-control group (*n* = 69). In comparison with the experimental group, the treatment group received extra administration of miR-146a mimics (20 nmol/mL, 0.25 mL/kg body weight) intranasally 30 min before the administration of pilocarpine. Because the pathogenic course of the lithium-pilocarpine model consists of an acute phase, a latent phase, and a chronic phase, the three groups were further divided as follows: acute-phase/latent-phase/chronic-phase experimental groups (APE/LPE/CPE, *n* = 23); acute-phase/latent-phase/chronic-phase treatment groups (APT/LPT/CPT, *n* = 23); and acute-phase/latent-phase/chronic-phase control groups (APC/LPC/CPC, *n* = 23). Subsequently, mice of the acute-phase, latent-phase, and chronic-phase groups were sacrificed at 2 h, 3 weeks, and 8 weeks after pilocarpine injection, respectively, to investigate the anti-inflammatory activities of miR-146a mimics in the lithium-pilocarpine model. Notably, 10 mice, 10 mice, and 3 mice per group were randomly used for miRNA/mRNAs quantitation (real-time qPCR), protein quantitation (Western blot, WB; enzyme linked immunosorbent assay, ELISA), and hippocampus staining (hematoxylin-eosin staining, HE; terminal deoxynucleotidyl transferase-mediated dUTP-biotin nick end labeling assay, TUNEL), respectively.

### 2.5. Behavior Observations

According to the groupings described above, 138 8-week-old postnatal mice (the experimental group and the treatment group) were subjected to the lithium-pilocarpine model. Within 1 h of the administration of pilocarpine, the percent of animals with no induced seizures, the latency to generalized convulsions, and the seizure severity were recorded to evaluate the potential of miR-146a mimics to improve seizure onset. In the case of generalized convulsions lasting more than 30 min, 3% chloral hydrate (2 mL/kg body weight, i.p.; Sigma-Aldrich, St. Louis, USA) was administered every 5 min until seizure cessation to reduce unexpected deaths among the experimental mice before their prespecified time of sacrifice. According to the severity of mice with epileptic seizures, the manifestations were classified into 5 levels: (1) twitching of facial muscle; (2) nodding of head; (3) unilateral forelimb with lifting or clonus; (4) bilateral forelimb with clonus as standing; (5) falling as standing or twisting.

### 2.6. Real-Time qPCR, ELISA, HE, and TUNEL Staining

All mice were sacrificed by decapitation under deep anesthesia (3% chloral hydrate, 10 mL/kg body weight, i.p.; Sigma-Aldrich, St. Louis, USA). Then, the hippocampus was quickly collected for the following experiments. (1) Real-time qPCR: total RNA was isolated in an RNase-free environment using an RNA extraction kit (Thermo Fisher Scientific, Waltham, USA), followed by reverse transcription using a First Strand cDNA Synthesis Kit (Thermo Fisher Scientific, Waltham, USA) in accordance with the manufacturer's instructions. The cDNA products were then amplified using a Light-Cycler 480 sequence detector system (Roche Applied Science, Penzberg, Germany), and the specific primers used in the real-time qPCR were as follows: IRAK-1 forward primer: 5′-CAGAACCACCACAGATCATCATC-3′ and reverse primer: 5′-AGGCTTCAATTCCAATAGCATCA-3′; TRAF-6 forward primer: 5′-AAAGCGAGAGATTCTTTCCCTG-3′ and reverse primer: 5′-ACTGGGGACAATTCACTAGAGC-3′. Finally, the relative expression levels of miR-146a and its target mRNAs (IRAK-1 and TRAF-6) were calculated using the 2^−ΔΔCT^ method. (2) ELISA: The concentrations of TNF-*α*, IL-1*β*, and IL-6 in the hippocampus were individually measured using enzyme linked immunosorbent assay (ELISA) kits (R&D Systems, Minneapolis, USA) according to the manufacturer's instructions. The absorbance was determined using an ELISA reader (Bio-Rad Laboratories, Hercules, USA), and the limits of detection of the ELISA kits for TNF-*α*, IL-1*β*, and IL-6 were 8 ng/mL, 5 pg/mL, and 2 pg/mL, respectively. (3) HE and TUNEL staining: the hippocampi were fixed in 4% paraformaldehyde overnight and then dehydrated in a graded ethanol series, embedded in paraffin, and cut into 4 *μ*m thick serial slices. Subsequently, these slices were deparaffinized and stained with HE for histopathological examination under light microscopy (Zeiss Axiovert S100TV, Jena, Germany). TUNEL staining was applied to determine in situ apoptosis in the slices using a TUNEL Apoptosis Detection Kit (Roche Applied Science, Penzberg, Germany) according to the manufacturer's instructions, followed by counterstaining with DAPI (Sigma-Aldrich, St. Louis, USA). Apoptotic indices of representative slices (apoptotic cells (cyan)/hippocampal cells (blue and cyan) in the merged pictures × 100%) were calculated using Image-Pro plus 6.0 (Media Cybernetics, Bethesda, USA).

### 2.7. Statistical Analysis

Displayed as the mean ± standard deviation (SD), the quantitative data were compared using Student's *t*-test. The enumeration data were compared using the Chi-squared test. The statistical analyses were performed with SPSS 19.0 (IBM, New York, USA), and a two-tailed *p* ≤ 0.05 was considered statistically significant. GraphPad Prism 5 (GraphPad, New York, USA) was applied to illustrate the findings. As no differences were observed among the APC/LPC/CPC groups, the APC/LPC/CPC groups were merged as the universal control (CON) to simplify Figures 3 and 4.

## 3. Results

### 3.1. Pilot Trials for Dynamic Changes in Relative miR-146a Levels and Effectiveness of the Lithium-Pilocarpine Protocol

**Figure 1:**
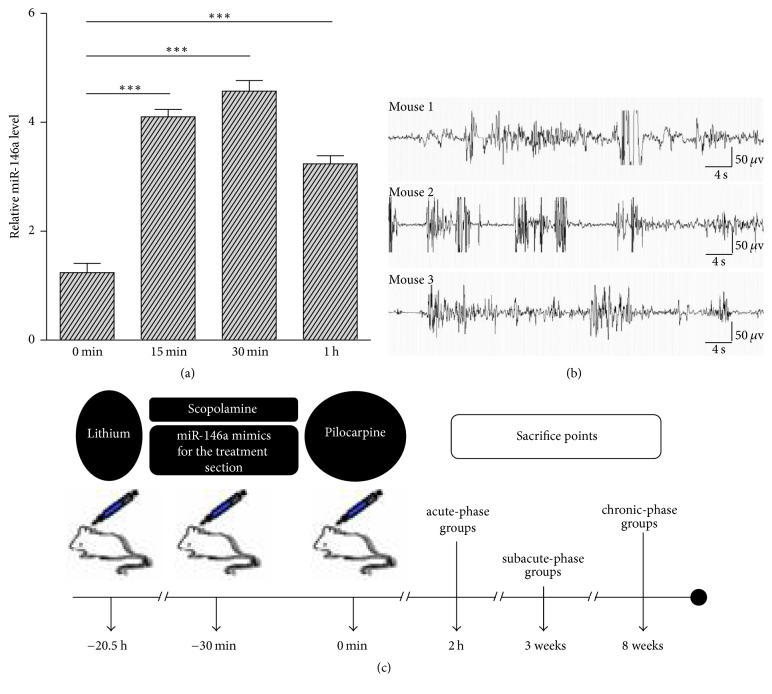
(a) Dynamic changes in relative miR-146a levels in the hippocampus after intranasal delivery of miR-146a mimics. Based on real-time qPCR, the relative miR-146a levels in the hippocampus were gradually upregulated at 15 min and 30 min after intranasal delivery of miR-146a mimics compared with the vehicle control sacrificed at 0 min and then began to decrease at 1 h compared with the 3.69-fold peak at 30 min. ^*∗∗∗*^*p* < 0.001. (b) Effectiveness of the lithium-pilocarpine protocol. While seizures began within 1 h after the administration of pilocarpine based on the Racine scale, tonic-clonic discharges were synchronously recorded in all three mice examined using the EEG/EMG acquisition system. (c) Graphic procedure of drug delivery and time of sacrifice in the subsequent experiments.

As shown in Figure 1(a), the dynamic changes in relative miR-146a levels in the hippocampus after intranasal delivery of miR-146a mimics indicated that miR-146a mimics could be delivered to the hippocampus via intranasal delivery, reaching a 3.69-fold peak at 30 min compared with the vehicle control sacrificed at 0 min. As shown in Figure 1(b), the effectiveness of the lithium-pilocarpine protocol used in the present study was further confirmed using EEG/EMG acquisition system, as well as the Racine scale. The drug delivery procedure and time of sacrifice of the mice in the subsequent experiments are described in detail in the Materials and Methods, which are summarized in Figure 1(c).

### 3.2. Behavioral Observations after Intranasal Delivery of miR-146a Mimics in the Acute Phase of the Lithium-Pilocarpine Model

**Figure 2:**
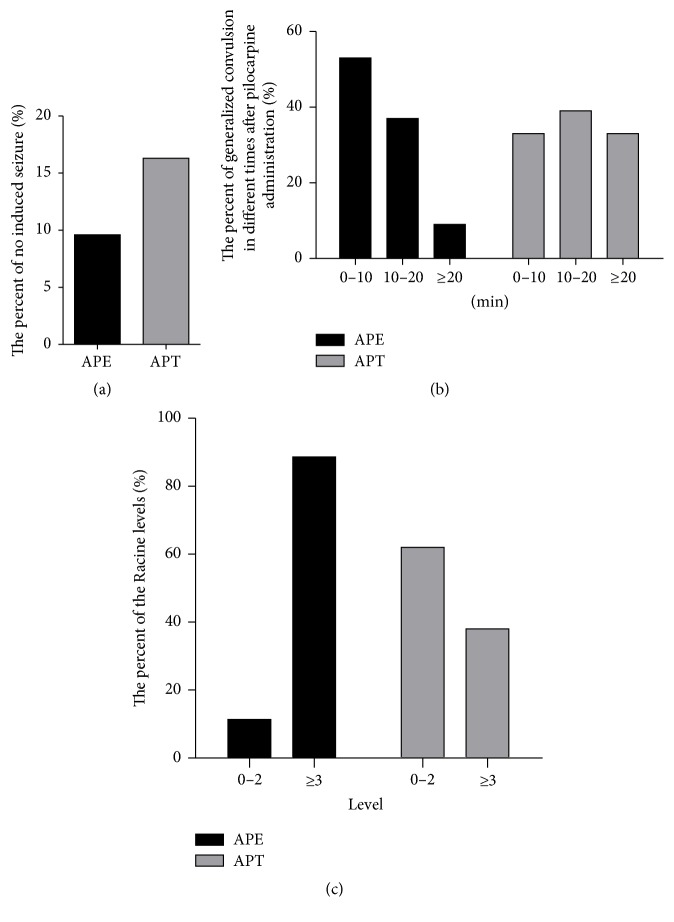
Behavioral observations within 2 h of pilocarpine administration. (a) Compared to the APE group, the percent of no induced seizures in the APT group increased by 6.7%; (b) the percentages of generalized convulsions were analyzed at different times after pilocarpine administration (0–10, 10–20, and ≥20 min) and revealed that the latency to generalized convulsions in the APT group was extended compared to the APE group; (c) the percentages of Racine levels (0–2 and ≥3) were analyzed based on the Racine scale and revealed that seizure severity in the APT group was reduced compared to the APE group.

As shown in Figure 2, the percentage of no induced seizures increased by 6.7% (a), the latency to generalized convulsion after pilocarpine administration was extended (b), and the seizure severity was reduced (c) in the APT group compared with the APE group. All these behavioral observations within 2 h after pilocarpine administration indicate that intranasal delivery of miR-146a mimics could improve seizure onset in the acute phase of the TLE model.

### 3.3. Relative Levels of miR-146a and Its Target mRNAs in the Acute Phase of the Lithium-Pilocarpine Model

**Figure 3:**
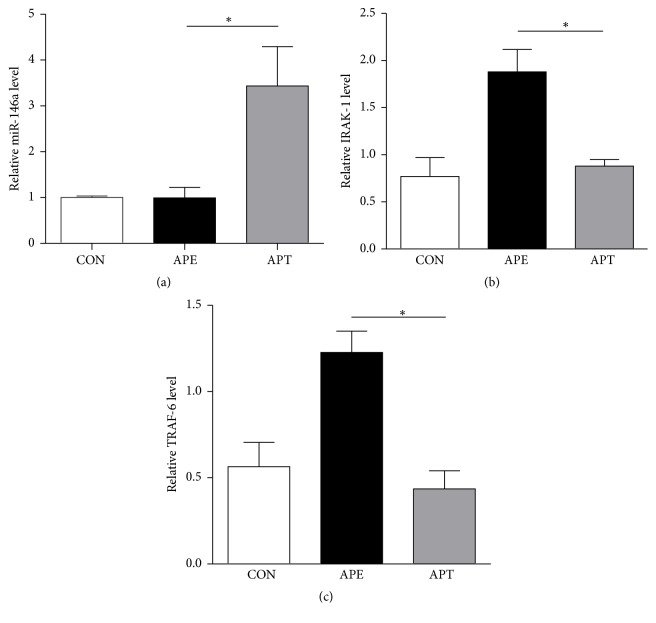
Relative levels of miR-146a and its target mRNAs 2 h after pilocarpine administration by real-time qPCR. (a) In comparison with the APE group, the relative level of miR-146a increased 3.42 times in the hippocampus of the APT group. ((b) and (c)) The expression of its target mRNAs IRAK-1 and TRAF-6 reduced 0.46 and 0.36 times in the hippocampus of the APT group compared with the APE group, respectively. Values are presented as the means ± SD (^*∗*^*p* < 0.05).

As shown in Figure 3, the relative level of miR-146a (a) significantly increased, yet the expression of its target mRNAs ((b) and (c)) was significantly reduced in the hippocampus of the APT group compared with the APE group. The trends of their relative levels 2 h after pilocarpine administration indicate that intranasal delivery of miR-146a mimics could upregulate miR-146a concentration in the hippocampus of the TLE model and, thereby, decrease the expression of its target mRNAs (IRAK-1 and TRAF-6).

### 3.4. Expression of Typical Inflammatory Modulators in the Acute Phase of the Lithium-Pilocarpine Model

**Figure 4:**
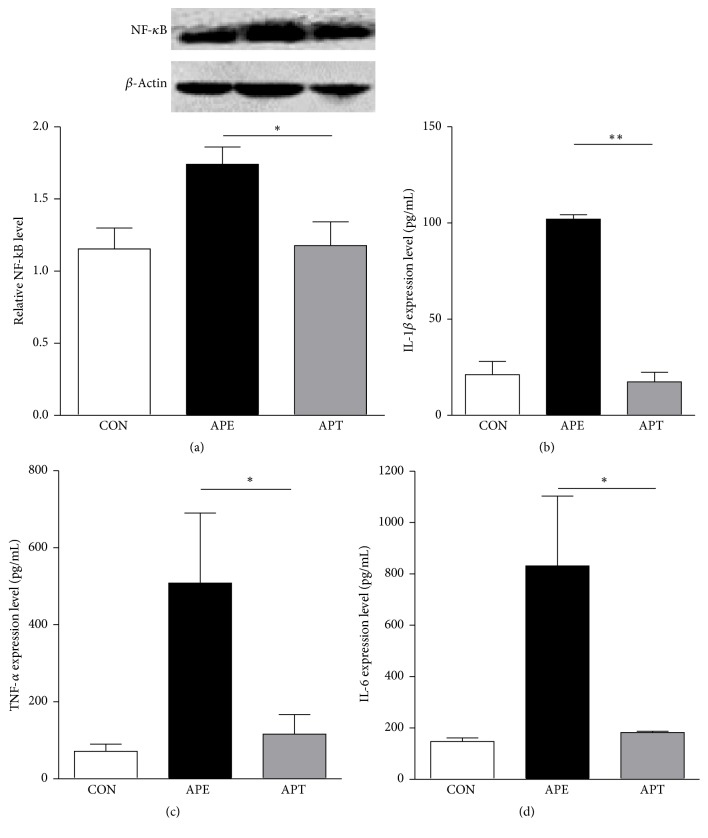
Expression of typical inflammatory modulators 2 h after pilocarpine administration. (a) The relative NF-*κ*B level reduced 0.68 times in the hippocampus of the APT group compared to the APE group according to real-time qPCR and confirmed by WB; ((b)–(d)) the expression of TNF-*α*, IL-1*β*, and IL-6 reduced 0.22, 0.17, and 0.23 times in the hippocampus of the APT group compared to the APE group, respectively. Values are presented as the means ± SD (^*∗*^*p* < 0.05; ^*∗∗*^*p* < 0.01).

As shown in Figure 4, the relative level of NF-*κ*B was demonstrated to be lower in the hippocampus of the APT group than in the APE group based on real-time qPCR, which was further confirmed by means of WB (a). Moreover, the expression levels of TNF-*α*, IL-1*β*, and IL-6 were also lower in the hippocampus of APT group than the APE group ((b)–(d)). All these consistent results indicate that intranasal delivery of miR-146a mimics significantly inhibits inflammatory activities in the acute phase of the TLE model.

### 3.5. Protective Effects on the Hippocampus in the Acute Phase of the Lithium-Pilocarpine Model

**Figure 5:**
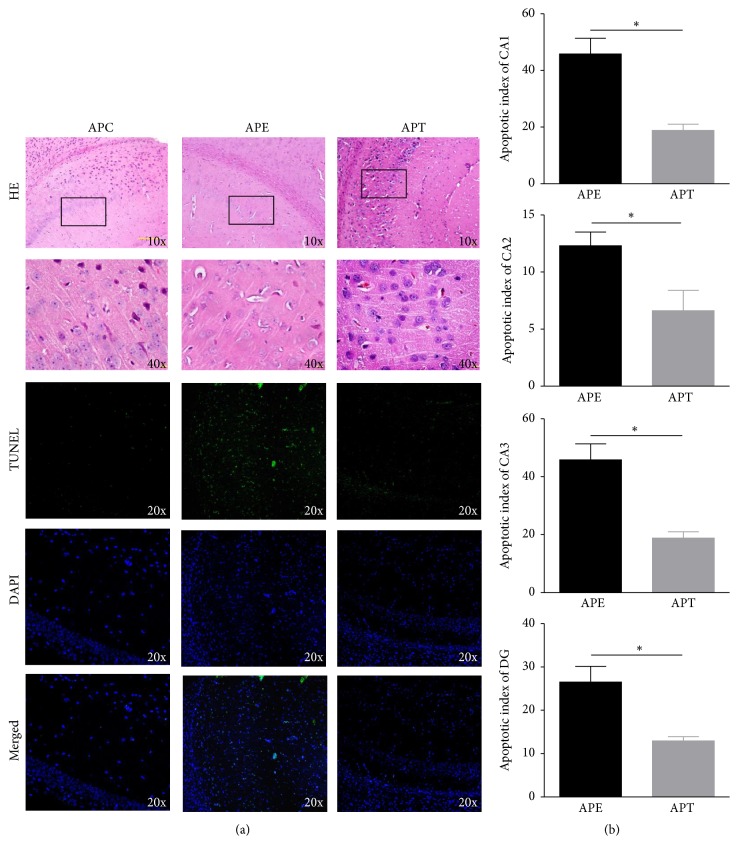
Protective effects of miR-146a mimics on the hippocampus 2 h after pilocarpine administration. (a) Representative HE-stained slices showed that the hippocampal nuclei (blue) almost disappeared in the APE group but were markedly stained in the APT group; representative TUNEL- and DAPI-stained slices showed all hippocampal cells (DAPI in blue) and significant apoptosis of hippocampal cells (TUNEL in green, merged TUNEL and DAPI in cyan) in the APE group, but no visible apoptosis was observed in the APT group. (b) To prevent manual mistakes, the apoptotic indices were calculated using Image-Pro plus 6.0 (Media Cybernetics, Bethesda, USA), which were consistently lower in all regions of the hippocampus (CA1, CA2, CA3, and DG) of the APT group than the APE group.^*∗*^*p* < 0.05.

As shown in Figure 5, the APE group underwent structural breakdown (shown by HE staining) and apoptosis of hippocampal cells (shown by TUNEL and DAPI staining), whereas intranasal delivery of miR-146a mimics significantly alleviated hippocampal damage in the APT group (a). Furthermore, the apoptotic indices of hippocampal cells were analyzed in all regions of the hippocampus (CA1, CA2, CA3, and DG), which further confirmed a protective role of miR-146a mimics in improving hippocampal damage in the acute phase of the TLE model (b).

## 4. Discussion

Based on expression profiling of miRNAs in previous studies, hundreds of miRNAs have been found to be abnormally expressed in epileptic tissues [31], whereas only several miRNAs, such as miR-21, miR-146a, and miR-155, have been reported to function in the pathological process of epilepsy [32]. Recently, our team found that expression of the functional rs57095329 A allele elevated the levels of anti-inflammatory miR-146a and was associated with a reduced risk of seizure frequency in drug-resistant patients containing 95% TLE cases [26], which further urged us to evaluate the potential of miR-146a as a molecular target for seizure treatment in the lithium-pilocarpine model. As a result, we first demonstrated that intranasal delivery of miR-146a mimics could improve seizure onset and hippocampal damage in the acute phase of the TLE model.

As a topical treatment of nasal disorders, intranasal delivery was further developed by Fray in 1989 for treatment of neurological diseases (International Patent Pub number WO/1991/007947), which represents a practical, noninvasive route to bypass the blood-brain barrier (BBB) to transport therapeutic and/or diagnostic drugs to the central nervous system [33]. Using autoradiographs of [^125^I]-IGF-I, two specific passages of intranasal delivery to the brain were clarified as the peripheral olfactory and trigeminal systems [34]. Recently, upregulated miR-203 in the hippocampi of mouse and human epileptic brains is considered to abnormally target inhibitory synaptic receptors, resulting in epileptic activities, and Lee et al. further demonstrated a peak of miR-203 inhibitors 1 h after intranasal delivery and its therapeutic role in chronic epileptic mice [35]. To our knowledge, this could be the first evidence that sheds light on the potential of artificially synthesized miRNA agents to treat epileptic seizures via intranasal delivery. As an anti-inflammatory miR-146a, we first demonstrated its antiepileptic roles via intranasal administration of miR-146a mimics in the lithium-pilocarpine model, expecting to supply an inflammation-based approach to improve seizure treatment, as well as miR-203 inhibitors by inhibiting synaptic transmission.

Although miRNAs make up a group of pleiotropic modulators due to their multiple target genes, which are involved in a wide range of biological activities, miR-146a mainly acts as an anti-inflammatory mediator in the brain according to the previous studies [25, 36]. Iyer et al. first observed that the transfection of astrocytes with miR-146a inhibitors or mimics negatively regulated the expression levels of downstream target genes (IRAK-1 and TRAF-6) [25]. In addition, miR-146a expression in human glial cell cultures was induced by IL-1*β* and blocked by an IL-1*β* receptor antagonist, which indicates that miR-146a plays an anti-inflammatory role in astrocytes via the TLR pathway [25]. Furthermore, increased expression of miR-146a was reported in gliosis lesions of experimental and human TLE [25, 36], while these lesions characterized by astrocytic dysfunction represent a basic pathogenic symptom of TLE. In fact, astrocytes have been proved to be implicated in the process of epileptic seizures, such as in the synchronization of neuronal firing, seizure generation, or spread [37]. Moreover, several membrane channels, receptors, and transporters in the astrocytic membrane have been found to be highly altered in epileptic brains [37]. This evidence indicates that miR-146a likely improves seizure activity by inhibiting the inflammatory activation of astrocytes and blocking the subsequent pathogenic alterations mentioned above.

As expected in our study, IRAK-1 and TRAF-6 were negatively regulated by intranasal delivery of miR-146a mimics, which is consistent with the increased level of miR-146a expression observed in the APT group. Similar to IRAK-1 and TRAF-6, the expressions of typical inflammatory modulators NF-*κ*B, TNF-*α*, IL-1*β*, and IL-6 were significantly downregulated in the APT group. According to the molecular pathway and the pathogenic symptoms of miR-146a in astrocytes and TLE [25, 36, 37], these findings strongly suggest that intranasal delivery of miR-146a mimics improves seizure onset and hippocampal damage in the acute phase of the TLE model via the TLR pathway. Nevertheless, miR-146a has been proven to regulate a series of other pathogeneses in non-brain cells and non-epilepsy diseases, such as immune disorders [38, 39], cancer [40, 41], and autophagy [42, 43], but whether these pathogeneses also function in the brains of TLE patients remains to be identified.

Certain limitations should be addressed in the present study. In addition to the acute phase, we explored the effects of miR-146a mimics in latent and chronic phases. We observed no extended protective effect with the recovery of miR-146a levels and no additional side effects, such as behavioral changes or inflammatory injuries, and therefore these negative results are not shown. Although intranasal delivery of miR-146a mimics has been demonstrated to improve seizure onset and hippocampal damage in the acute phase of the TLE model, its effects on spontaneous seizures in the chronic phase of the TLE model should still be explored, because the nature of spontaneous seizures is more similar to human TLE. In addition, we observed a 3.69-fold peak in miR-146a 30 min after intranasal delivery, at which time pilocarpine was administered, expecting to maximize the effect of the miR-146a mimics on epileptic seizures. It is possible that modification of the protocol, such as other time points of pilocarpine administration after intranasal delivery and other final volumes and dosages of miR-146a mimics, could not lead to the entirely same results.

In conclusion, our study is the first to demonstrate that intranasal delivery of miR-146a mimics can improve seizure onset and hippocampal damage in the lithium-pilocarpine-induced C57BL/6 mouse model, which provides additional inflammation-based clues for the development of novel TLE treatments.

## Supplementary Material

Representative TUNEL- and DAPI-stained slices in all four regions of the hippocampus (CA1, CA2, CA3, and DG) are available online for reference.
