# Supplementary material for: Intranasal Delivery of miR-146a Mimics Delayed Seizure Onset in the Lithium-Pilocarpine Mouse Model
Source: Mediators Inflamm. 2017 Jan 24;2017:6512620. doi: 10.1155/2017/6512620 (PMC5294386; doi:10.1155/2017/6512620)
Supplement: Supplementary file 1 — Representative TUNEL- and DAPI-stained slices in all four regions of the hippocampus (CA1, CA2, CA3, and DG) are available online for reference. [file 6512620.f1.doc]

**Hippocampus C1**

| **APC group** | | |
| --- | --- | --- |
| TUNEL | DAPI | Merged |
| 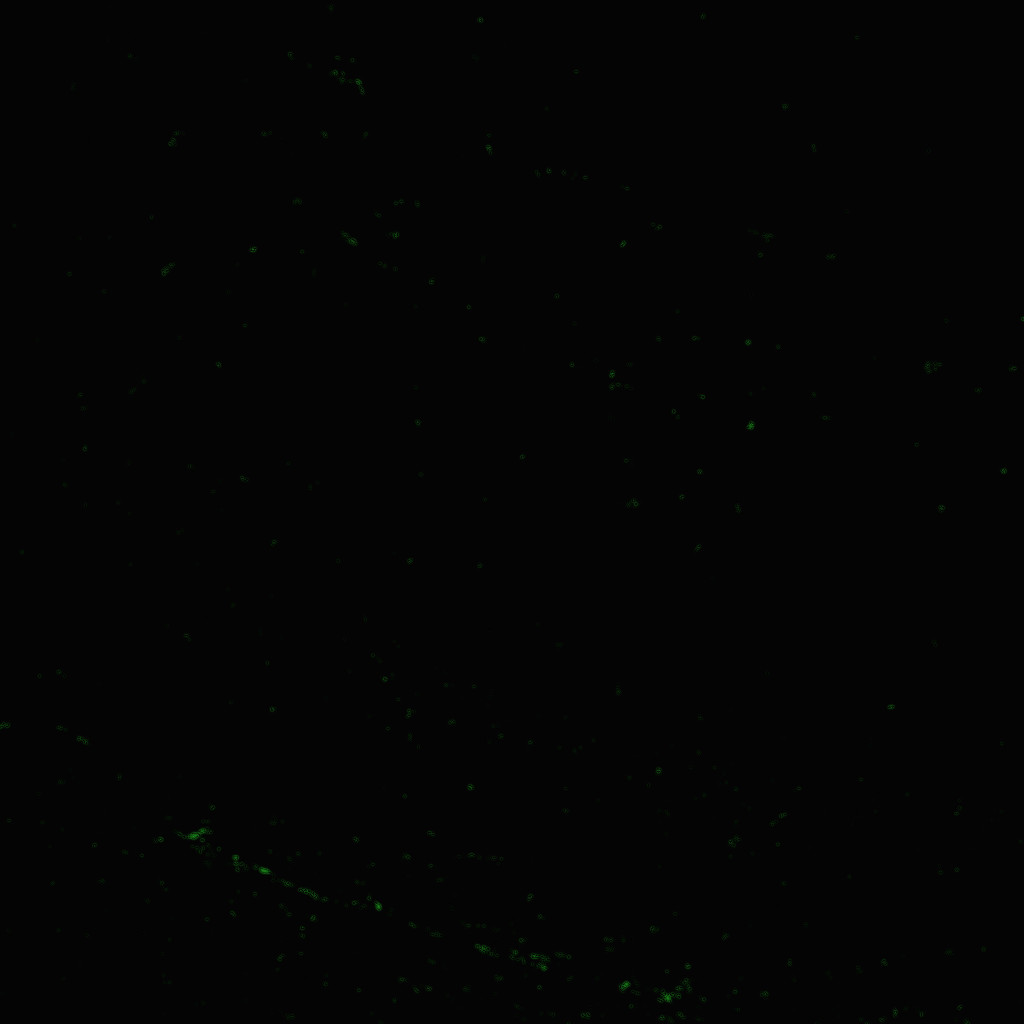 | 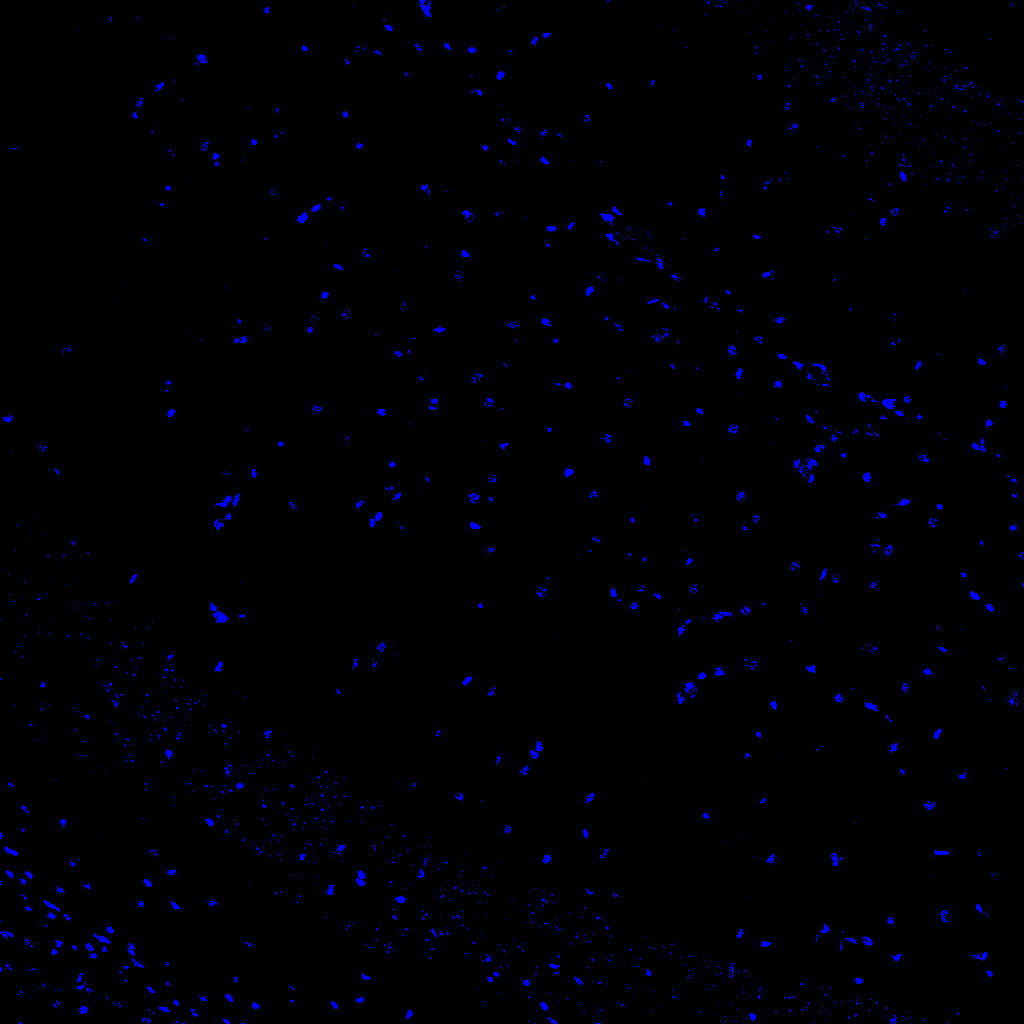 | 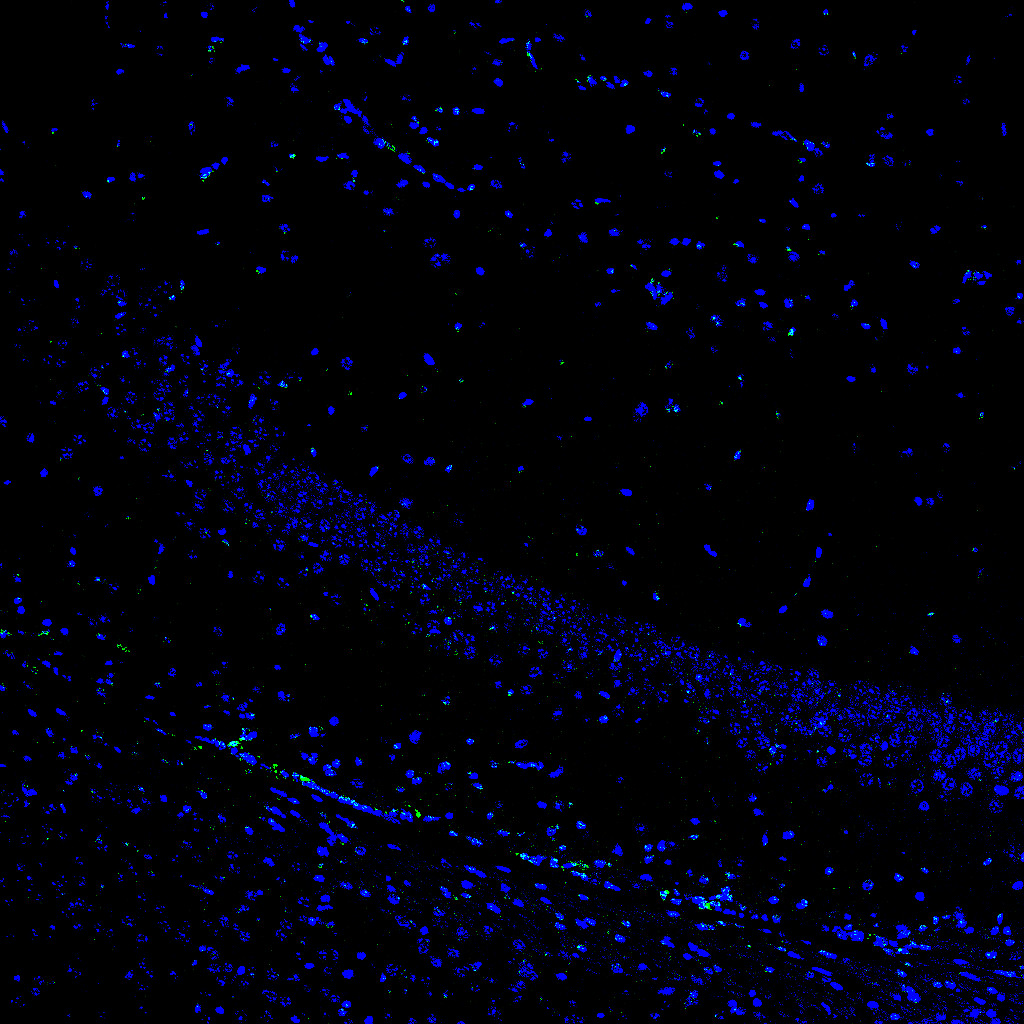 |
| **APE group** | | |
| TUNEL | DAPI | Merged |
| 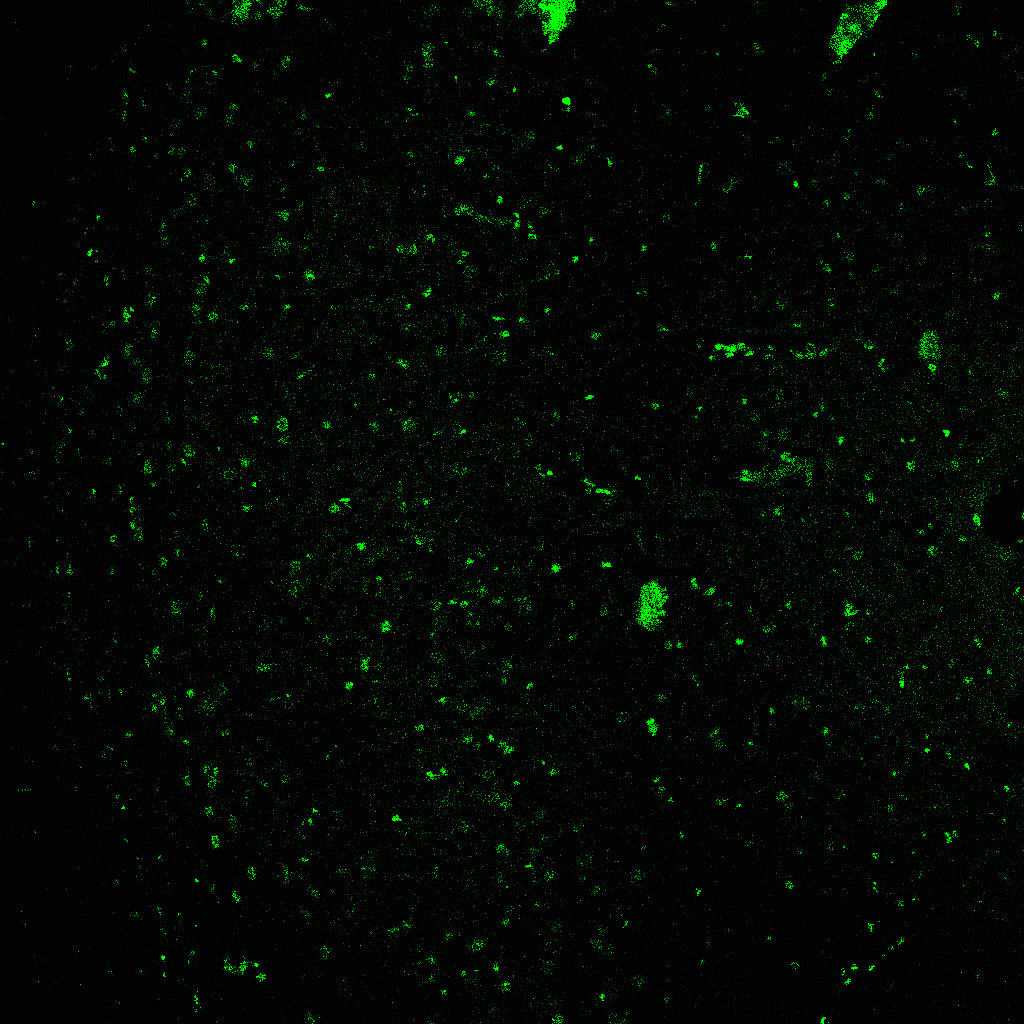 | 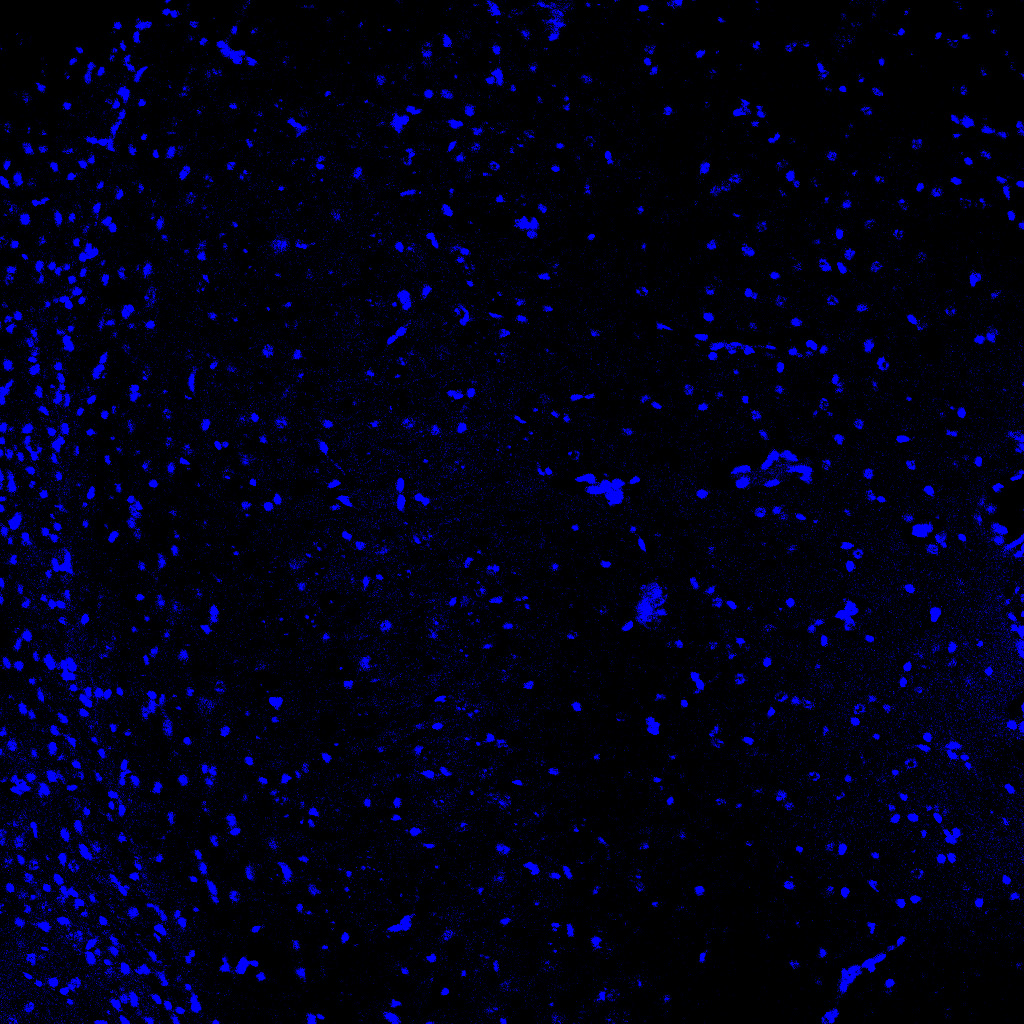 | 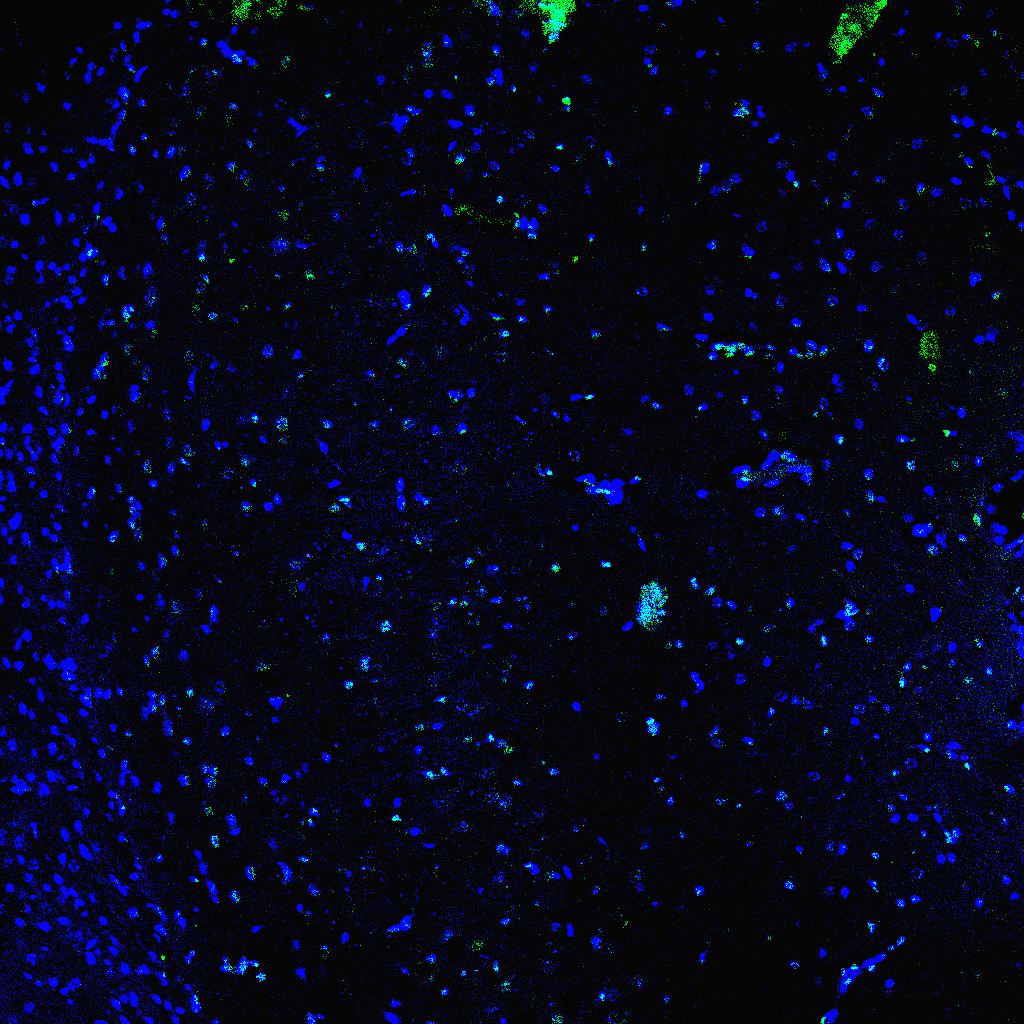 |
| **APT group** | | |
| TUNEL | DAPI | Merged |
| 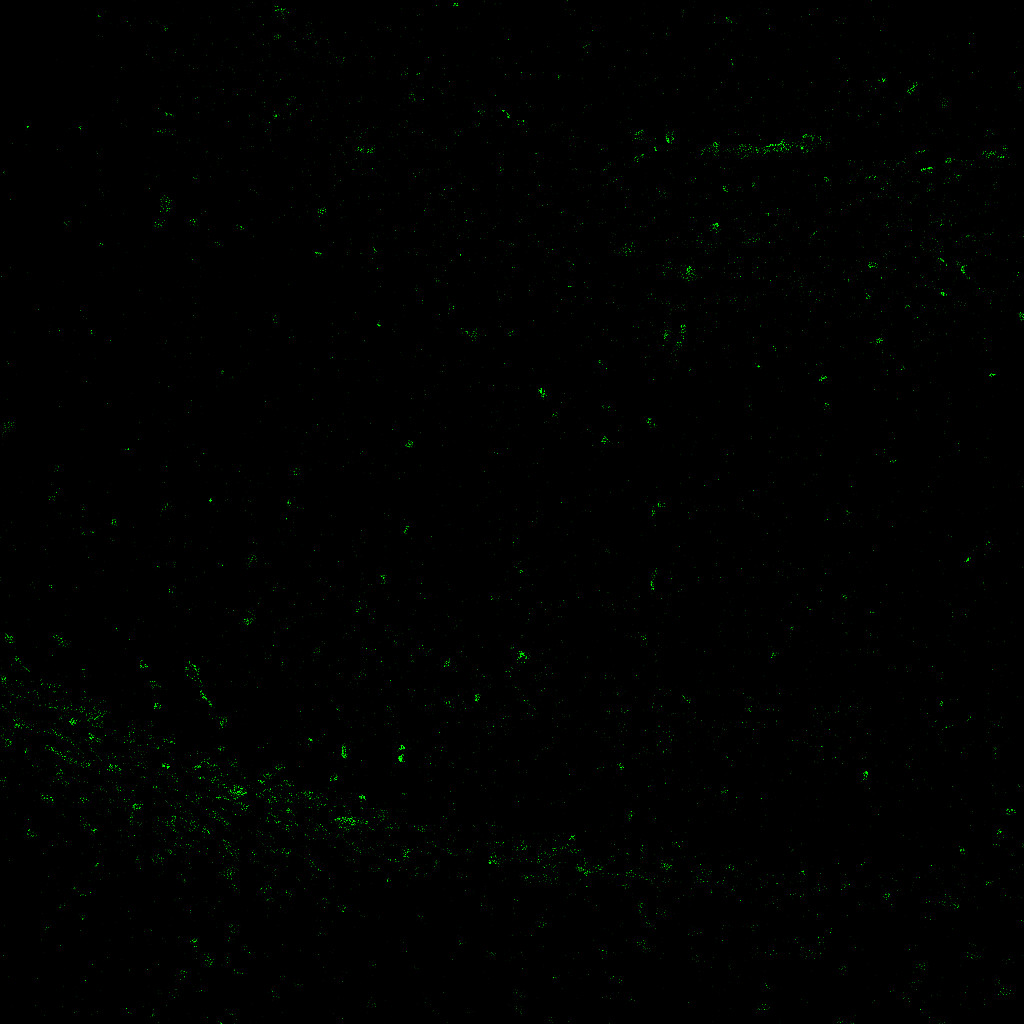 | 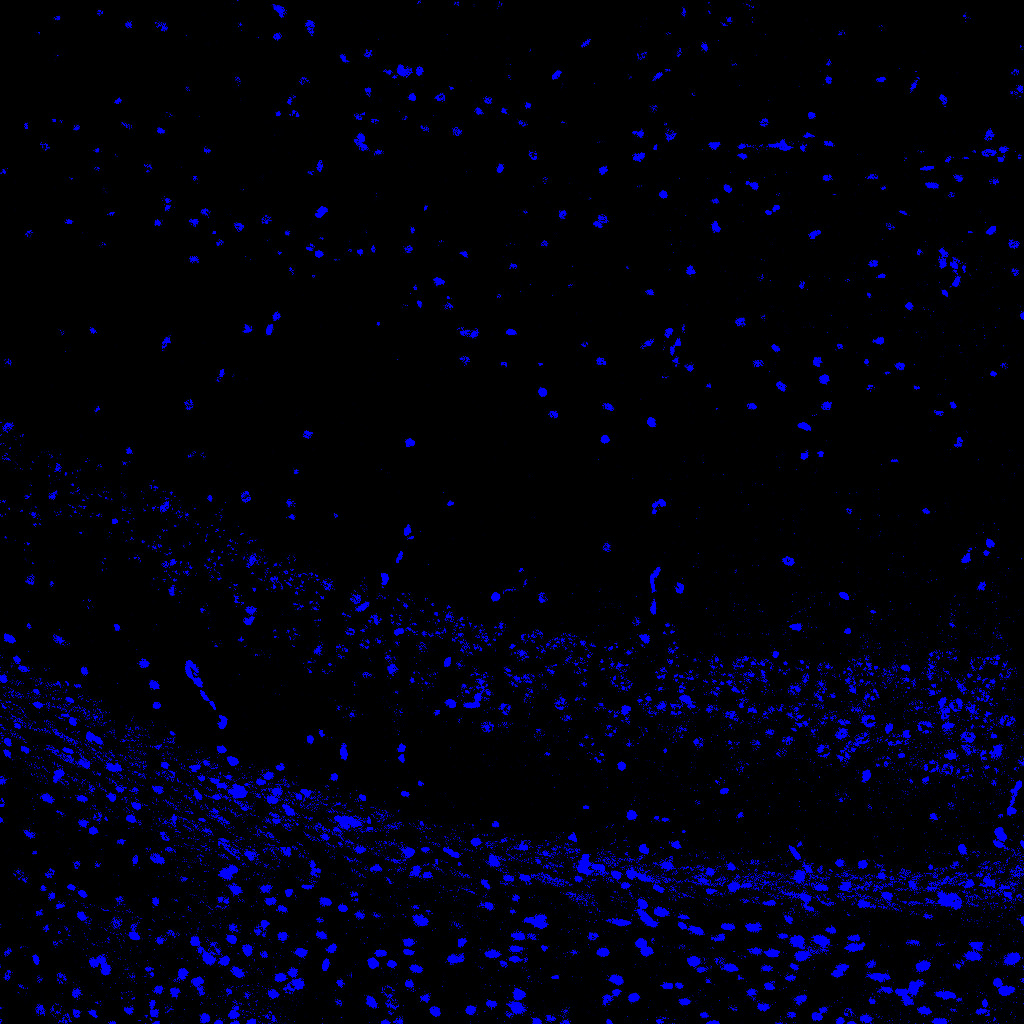 | 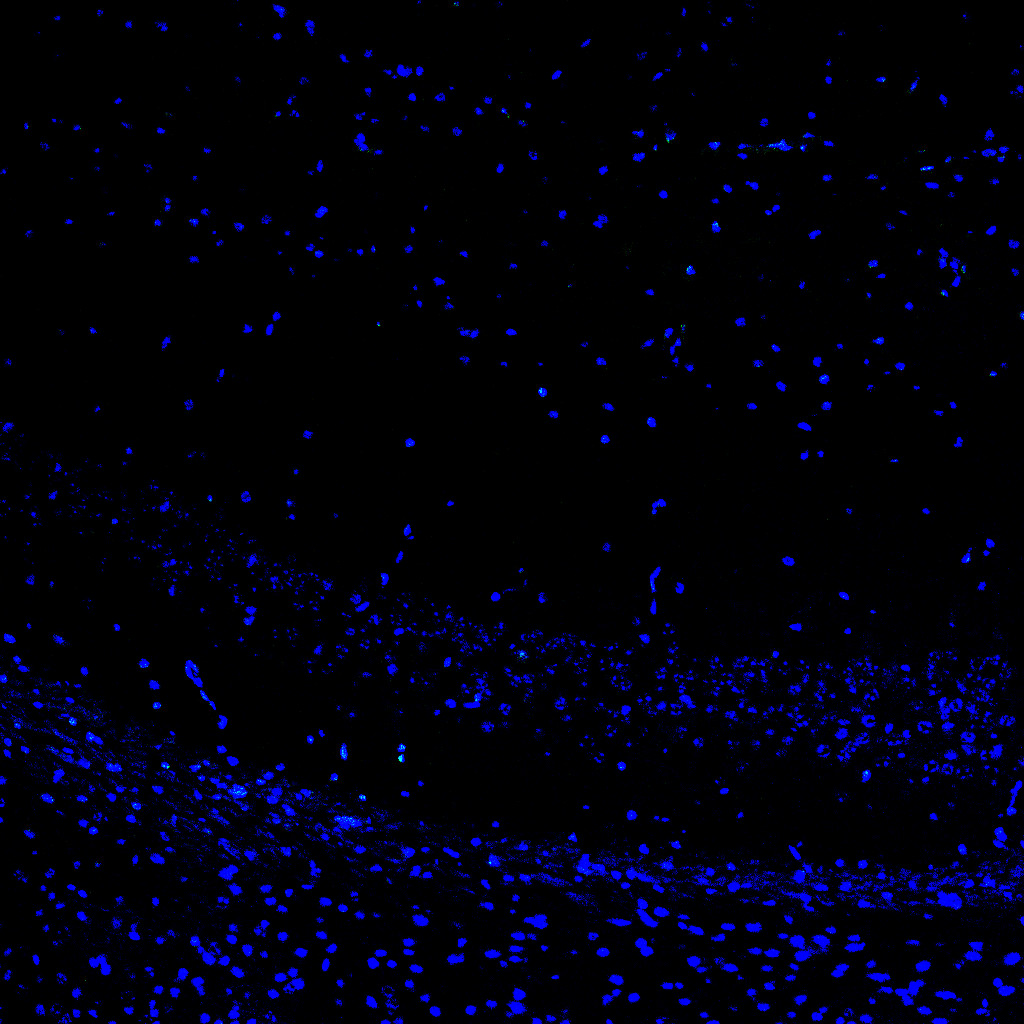 |

Supplementary Figure 1. The original image of TUNEL and DAPI staining in C1 regions of the hippocampus (20×).

**Hippocampus C2**

| **APC group** | | |
| --- | --- | --- |
| TUNEL | DAPI | Merged |
| 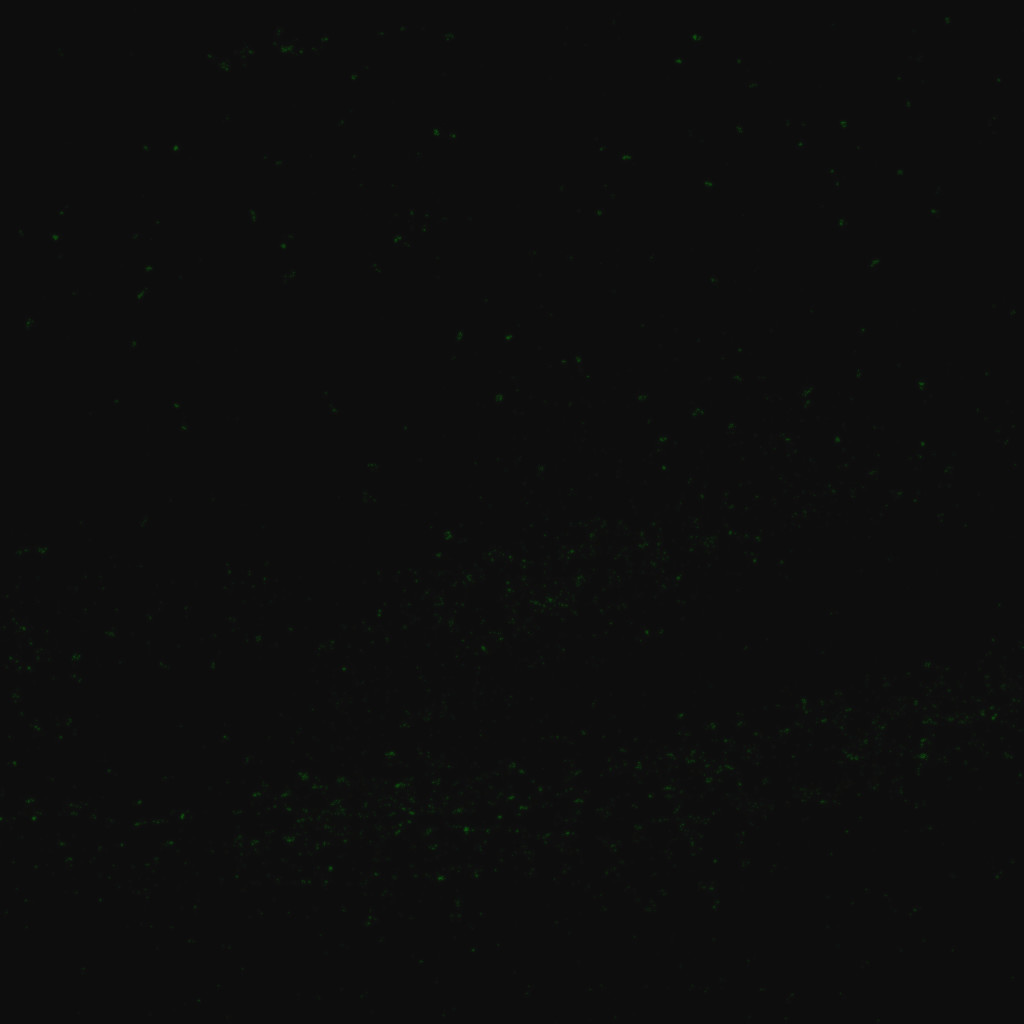 | 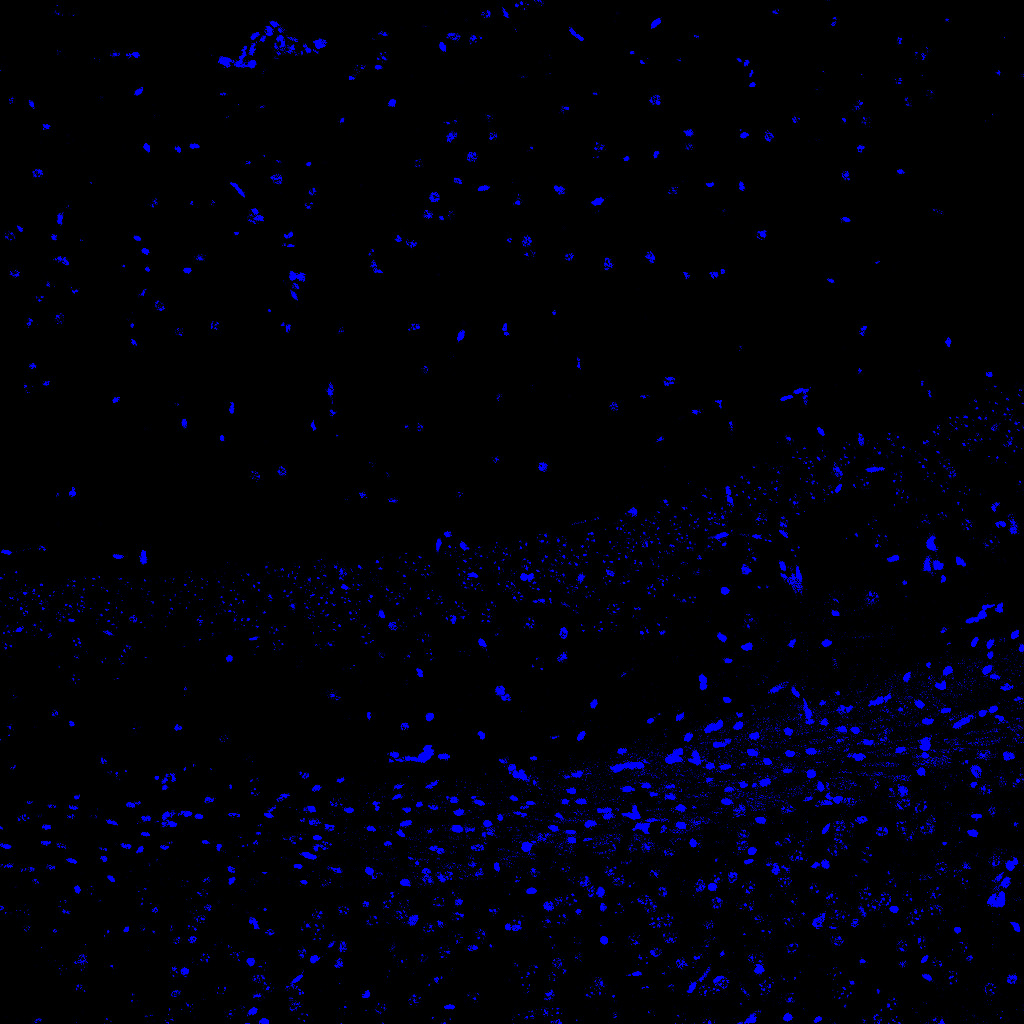 | 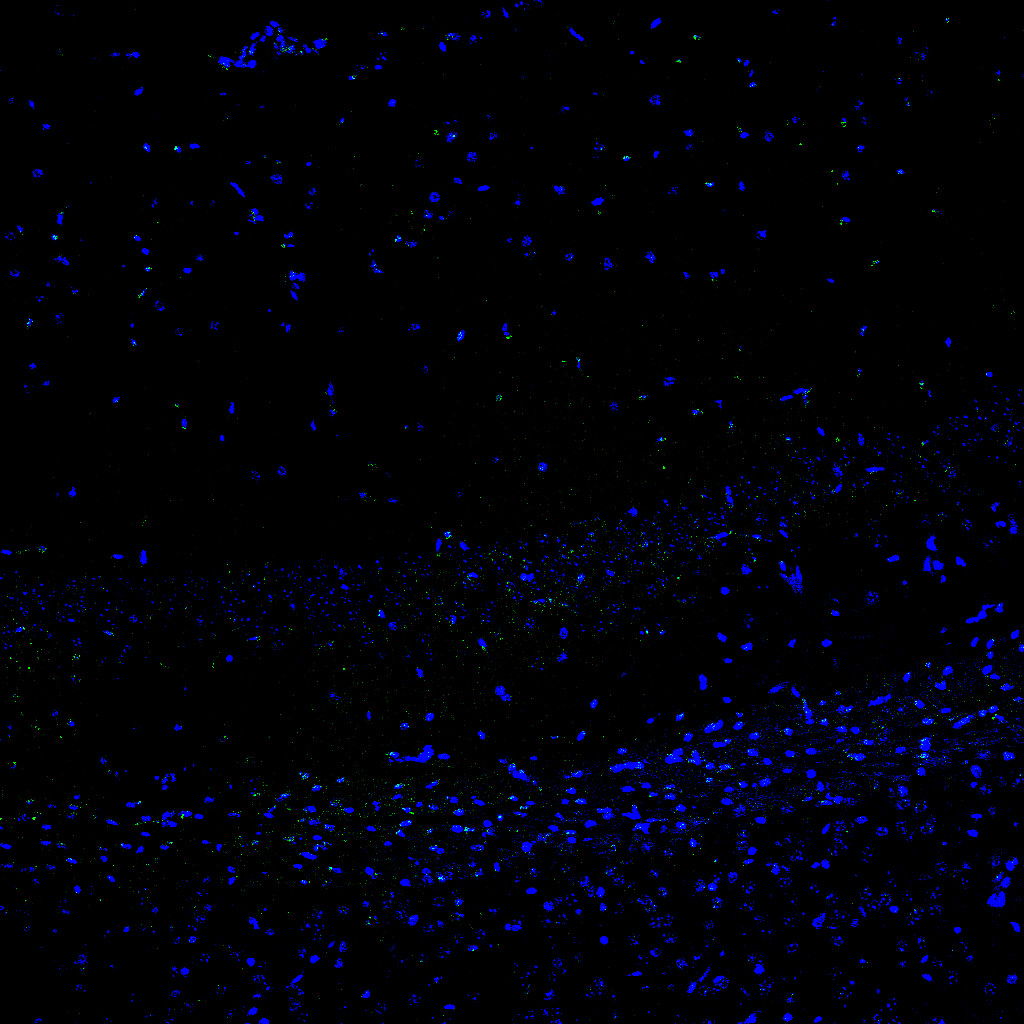 |
| **APE group** | | |
| TUNEL | DAPI | Merged |
| 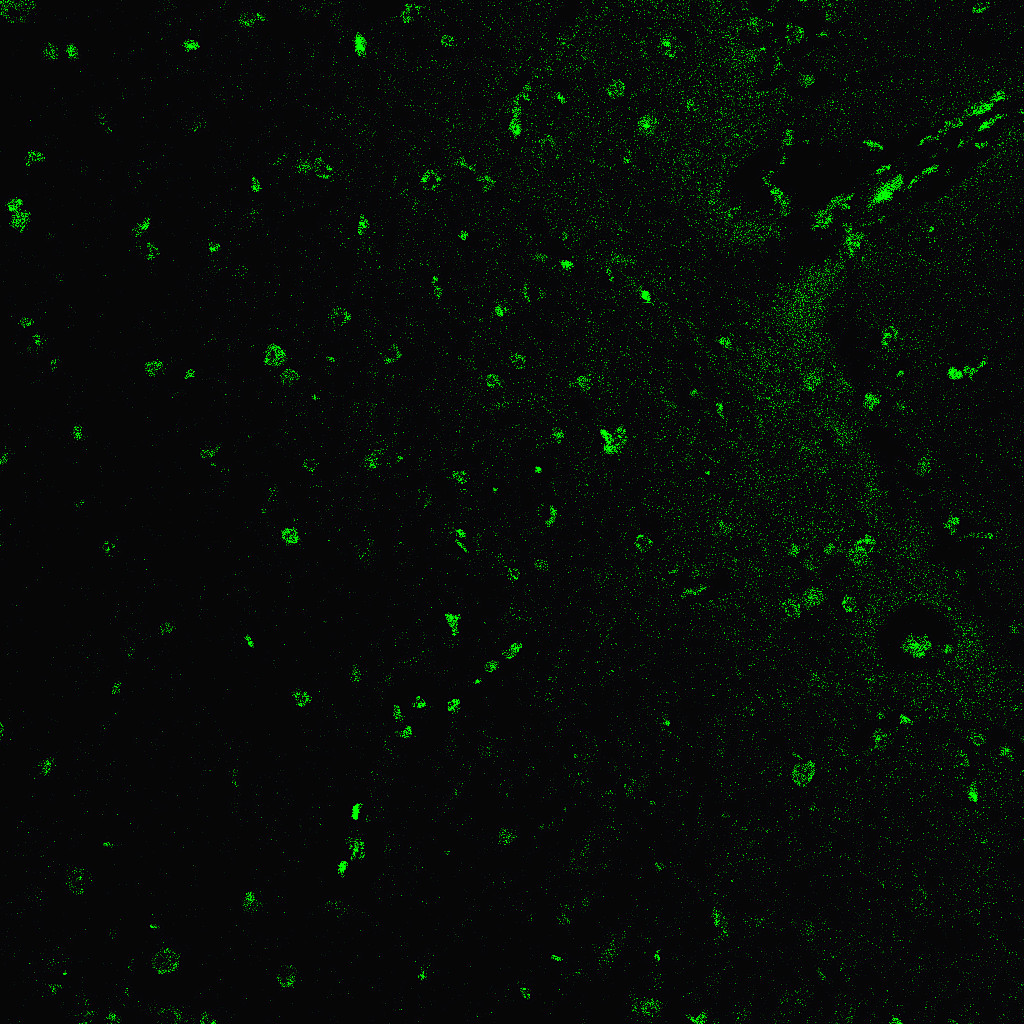 | 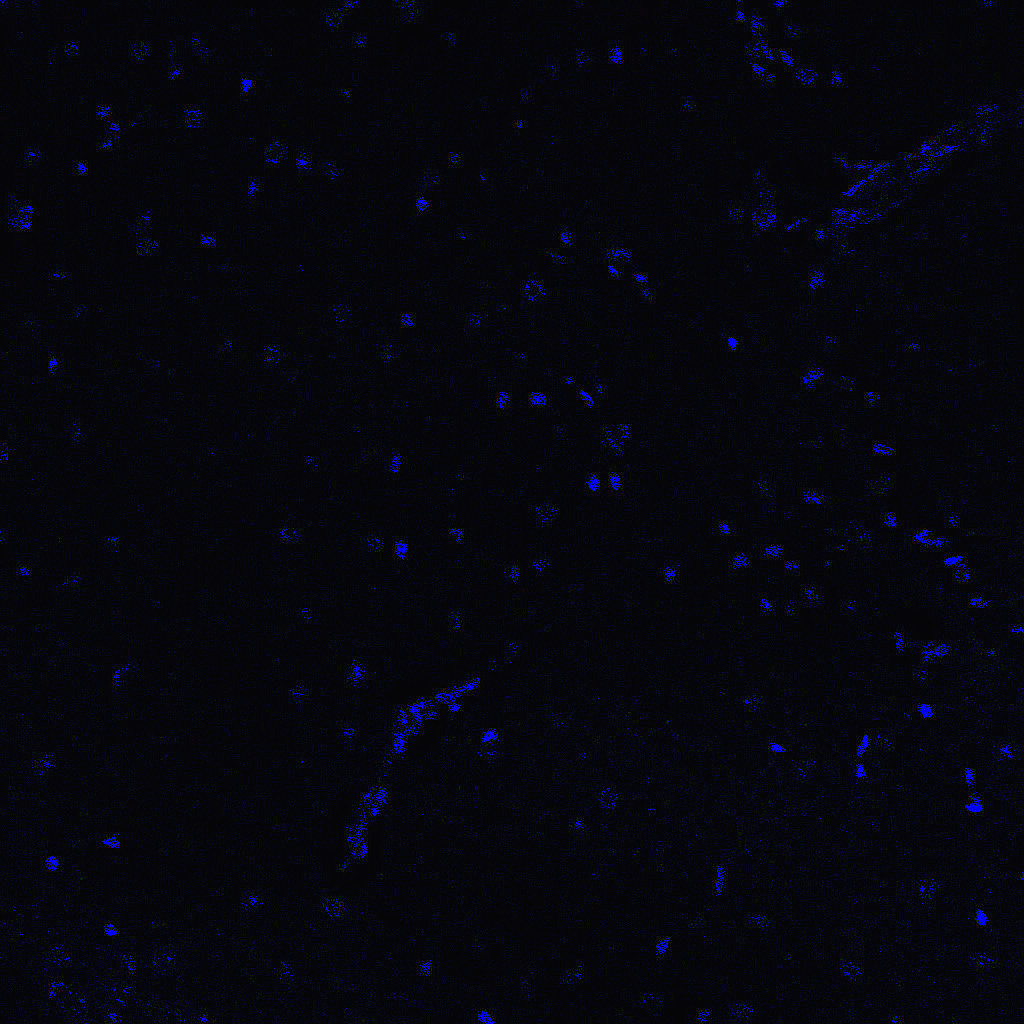 | 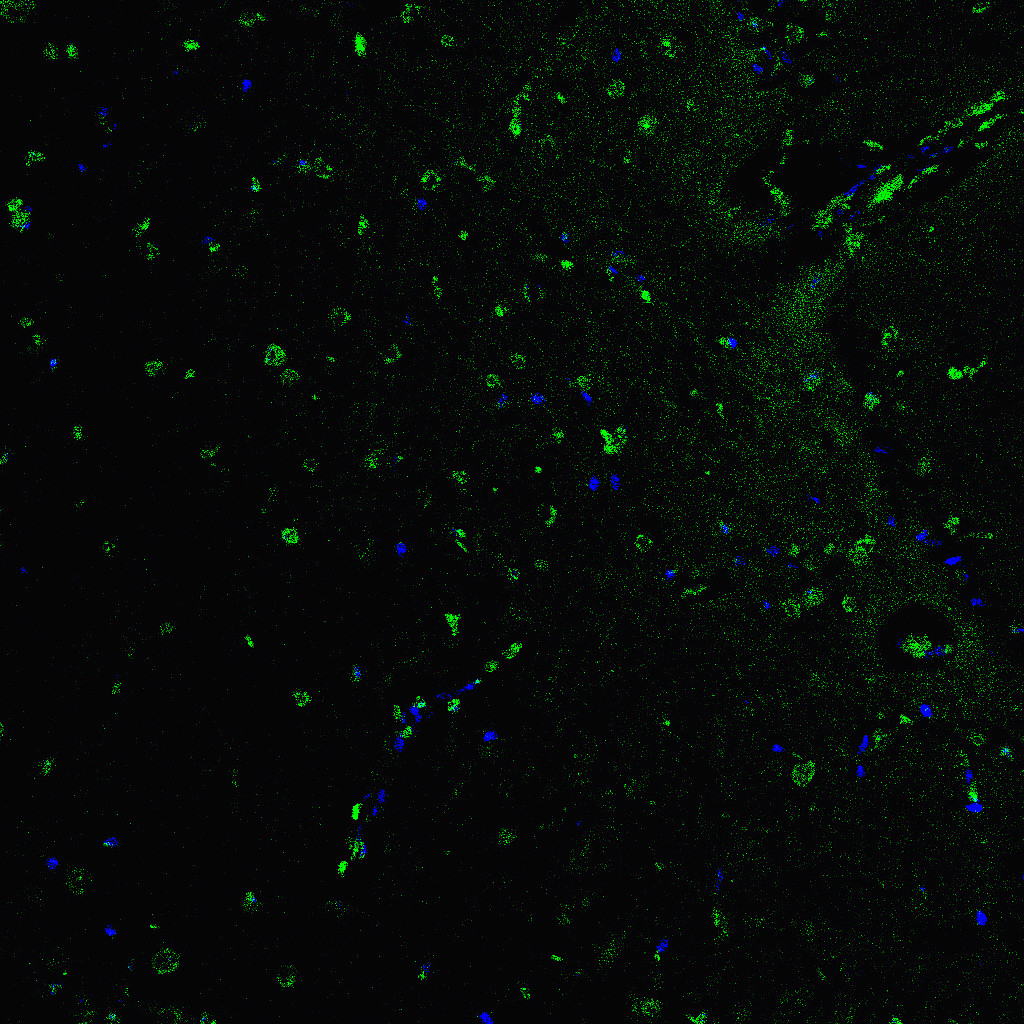 |
| **APT group** | | |
| TUNEL | DAPI | Merged |
| 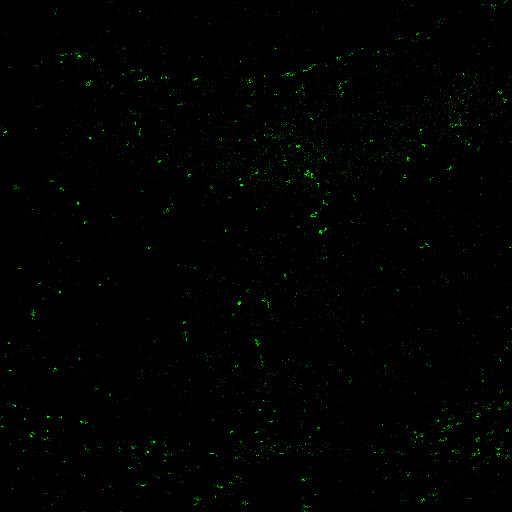 | 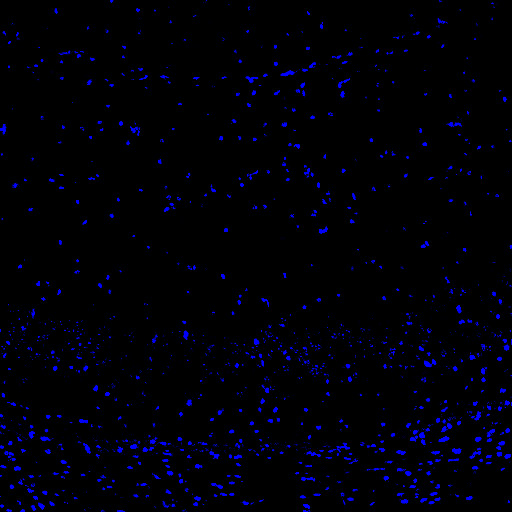 | 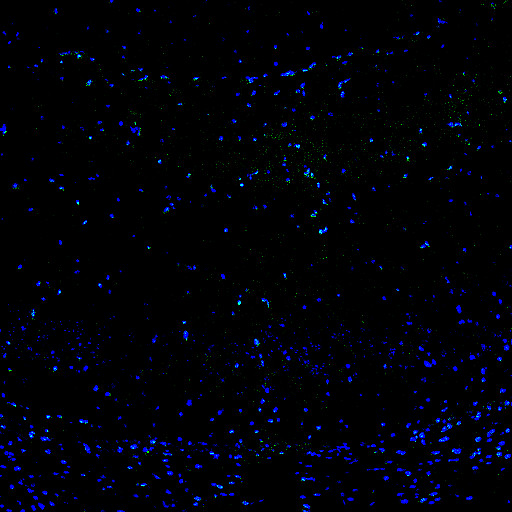 |

Supplementary Figure 2. The original image of TUNEL and DAPI staining in C2 regions of the hippocampus(20×).

**Hippocampus C3**

| **APC group** | | |
| --- | --- | --- |
| TUNEL | DAPI | Merged |
| 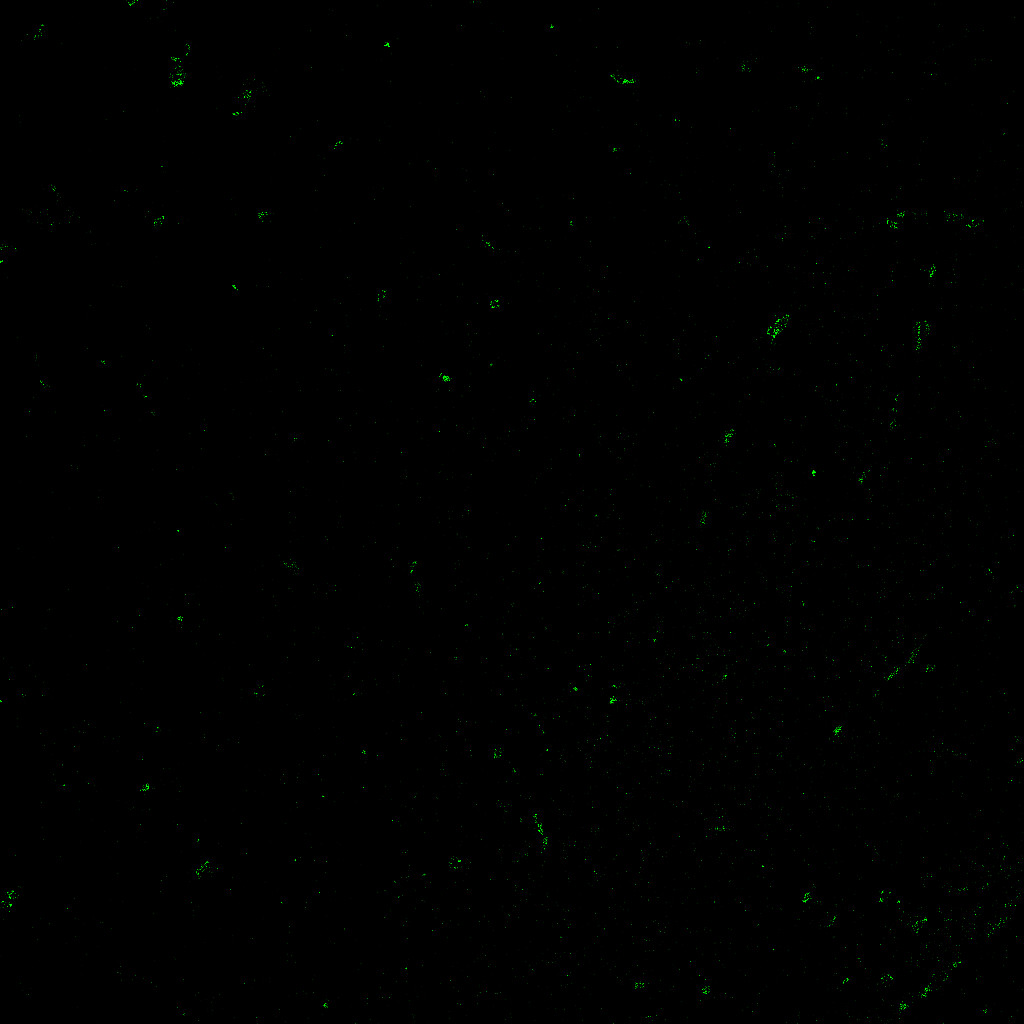 | 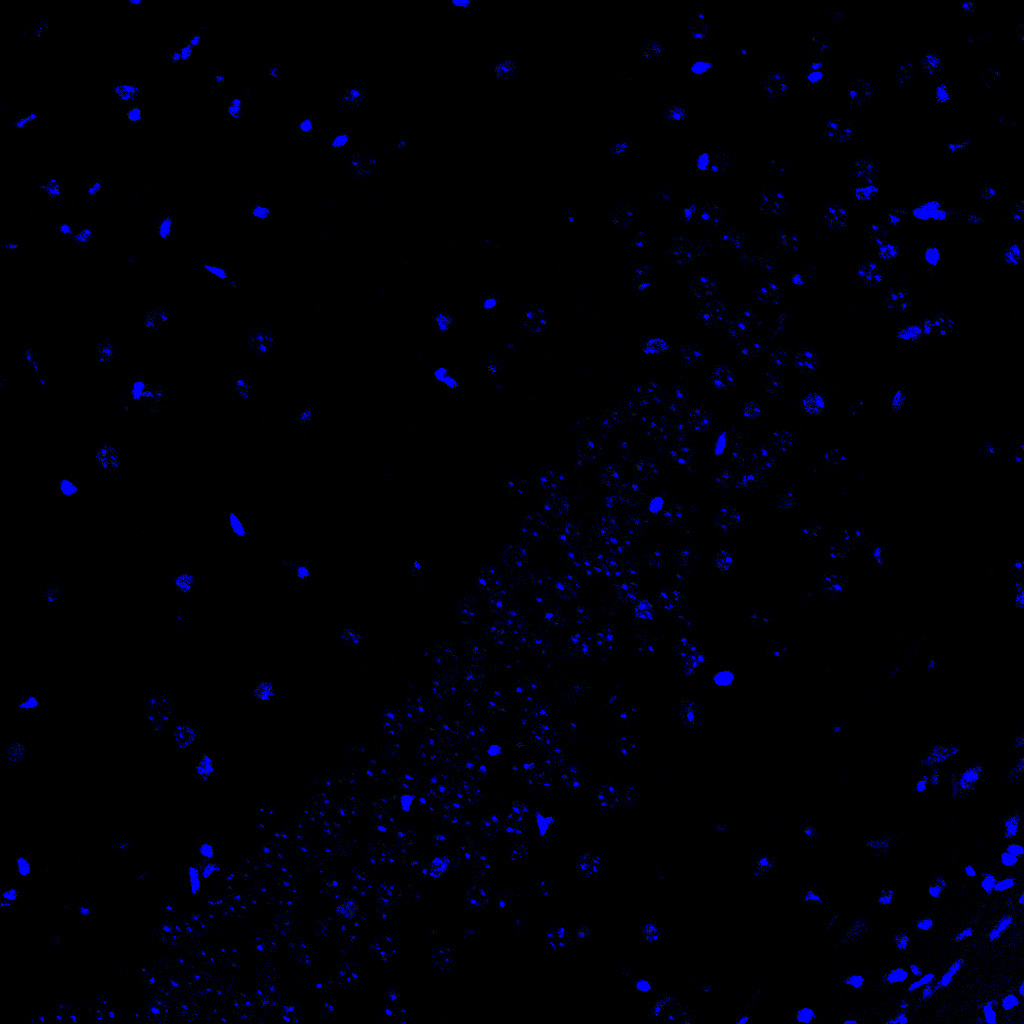 | 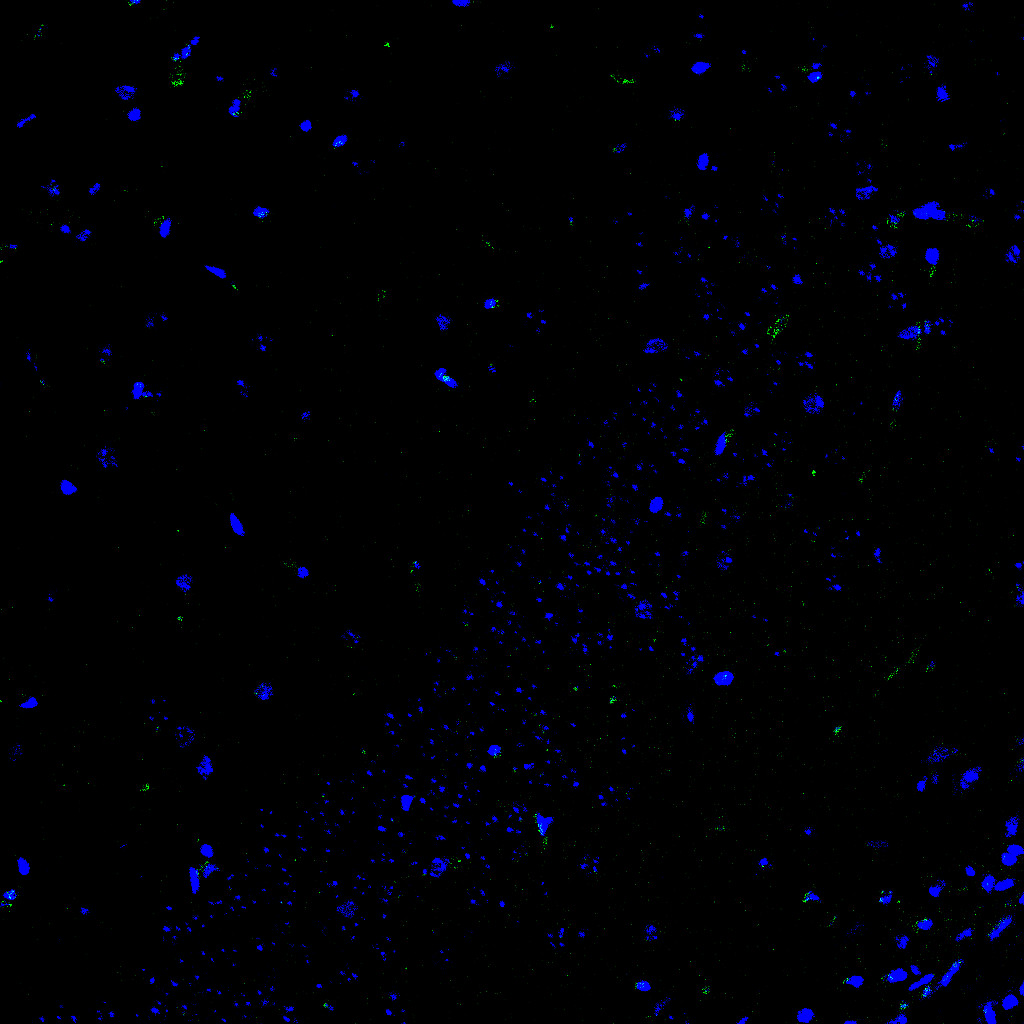 |
| **APE group** | | |
| TUNEL | DAPI | Merged |
| 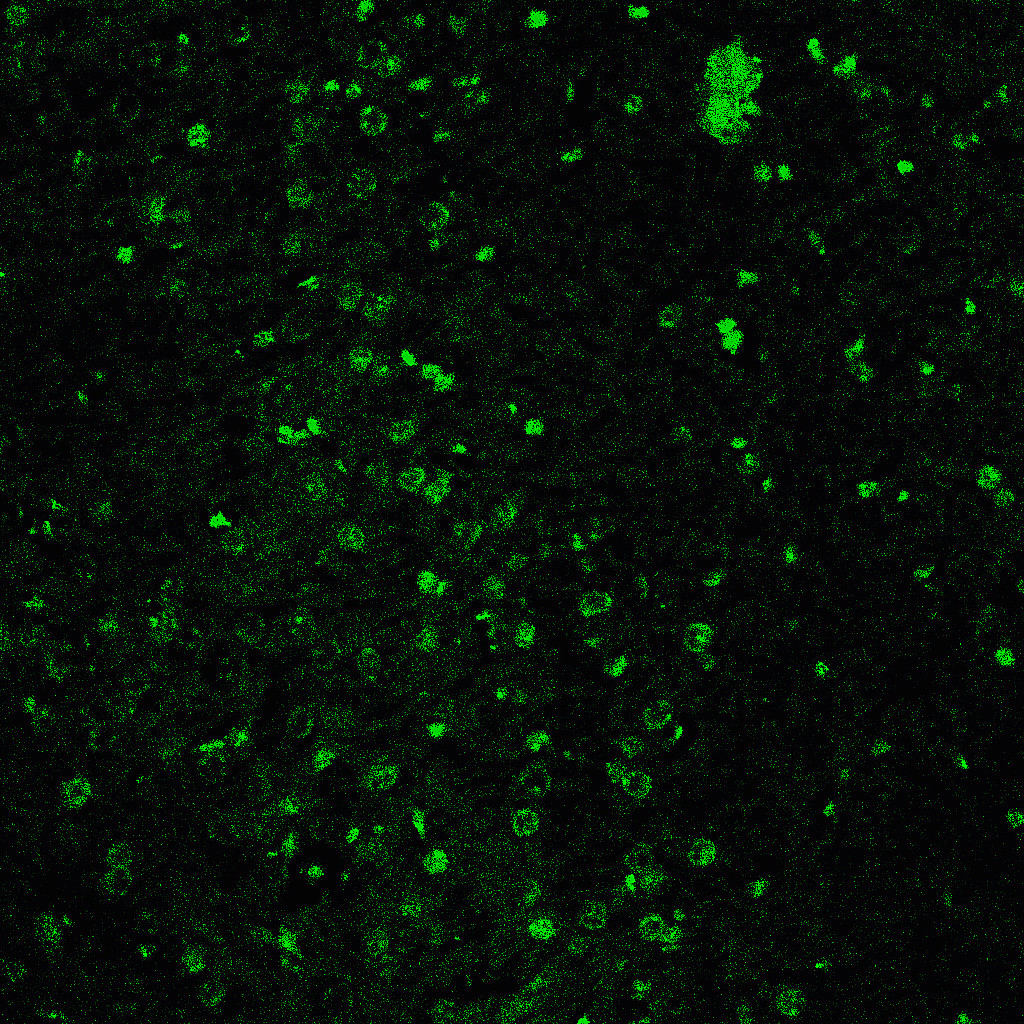 | 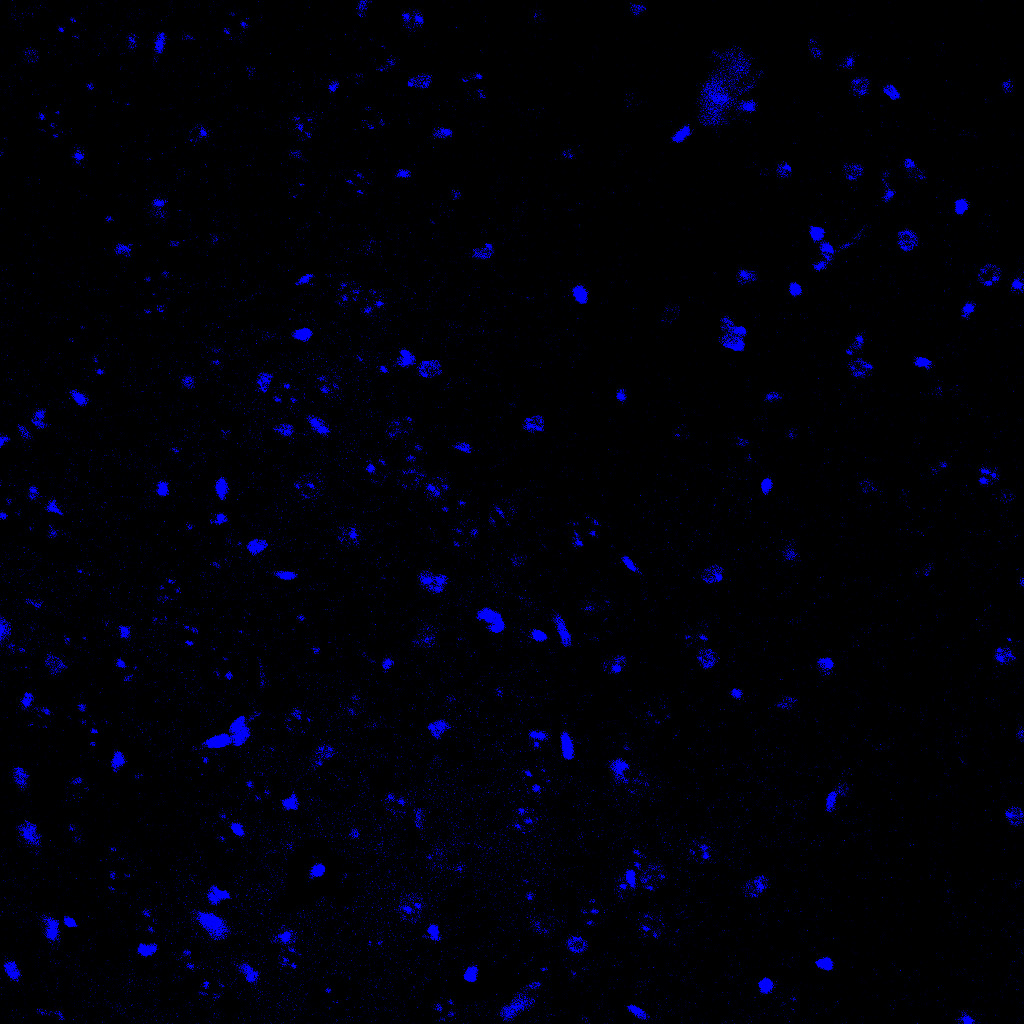 | 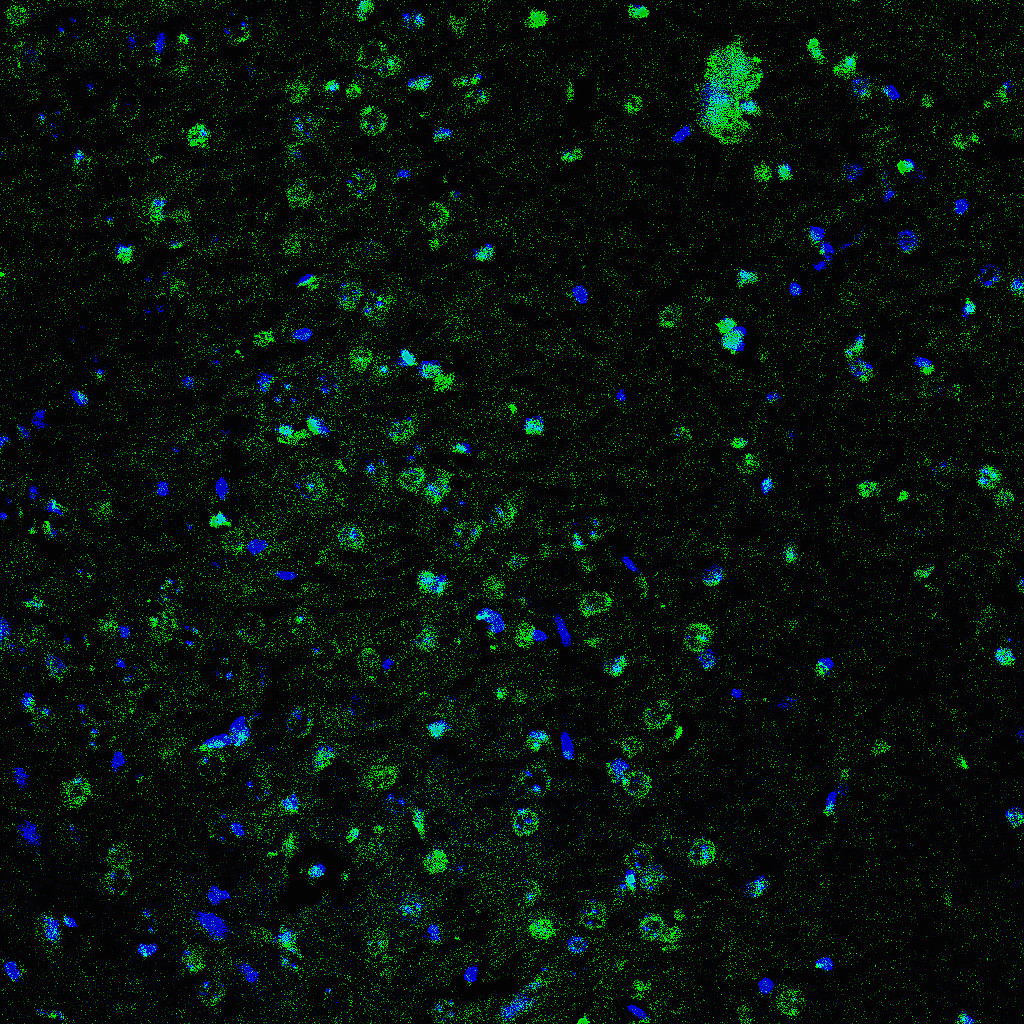 |
| **APT group** | | |
| TUNEL | DAPI | Merged |
| 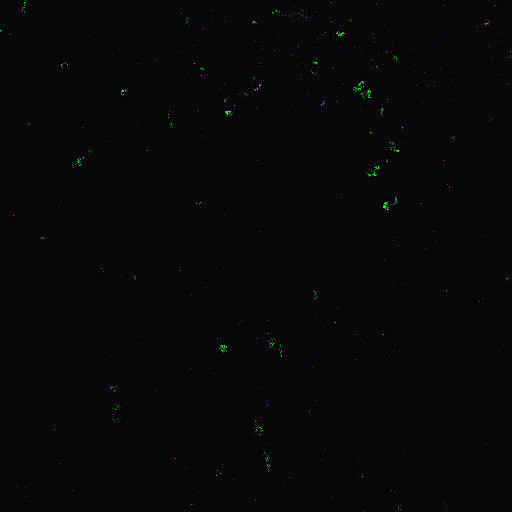 | 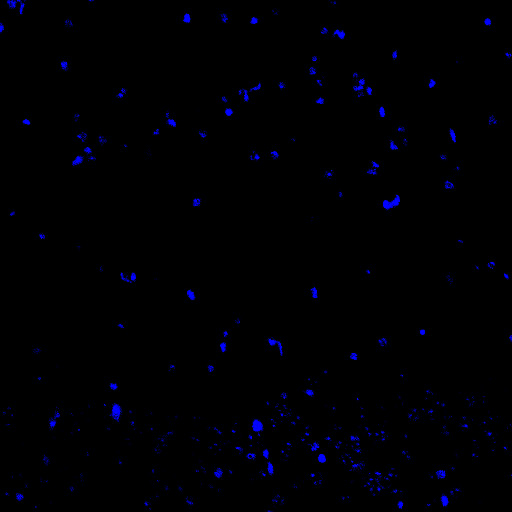 | 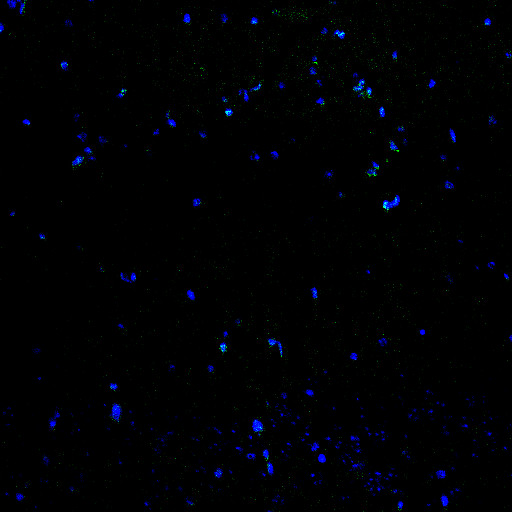 |

Supplementary Figure 3. The original image of TUNEL and DAPI staining in C3 regions of the hippocampus(20×).

**Hippocampus DG**

| **APC group** | | |
| --- | --- | --- |
| TUNEL | DAPI | Merged |
| 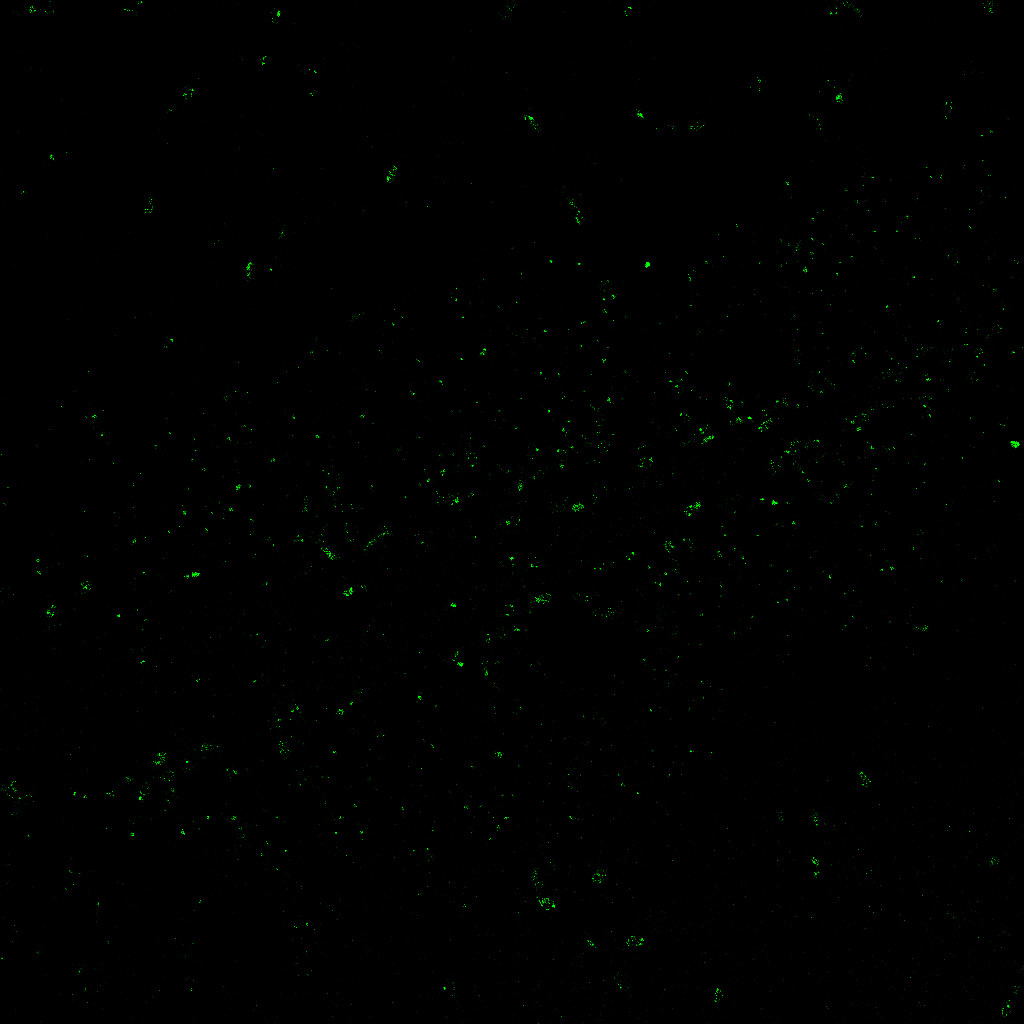 | 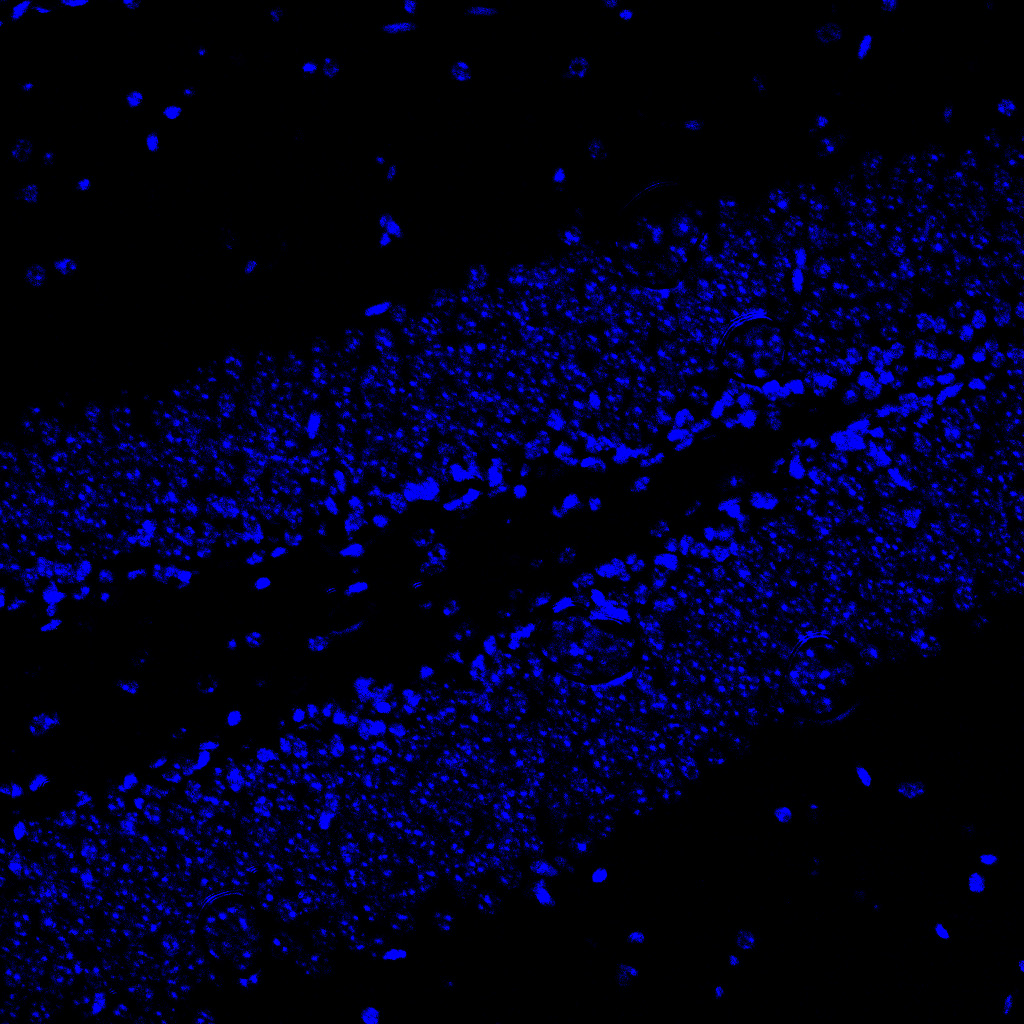 | 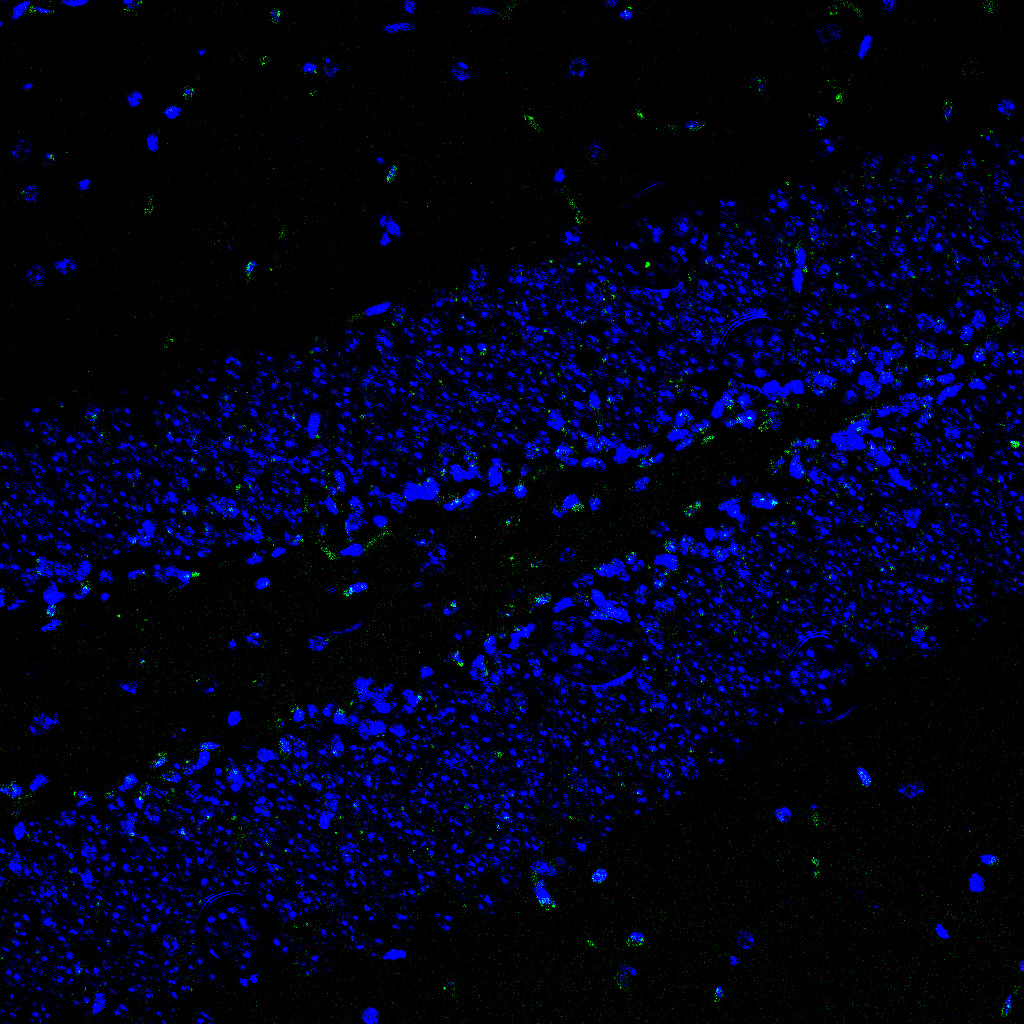 |
| **APE group** | | |
| TUNEL | DAPI | Merged |
| 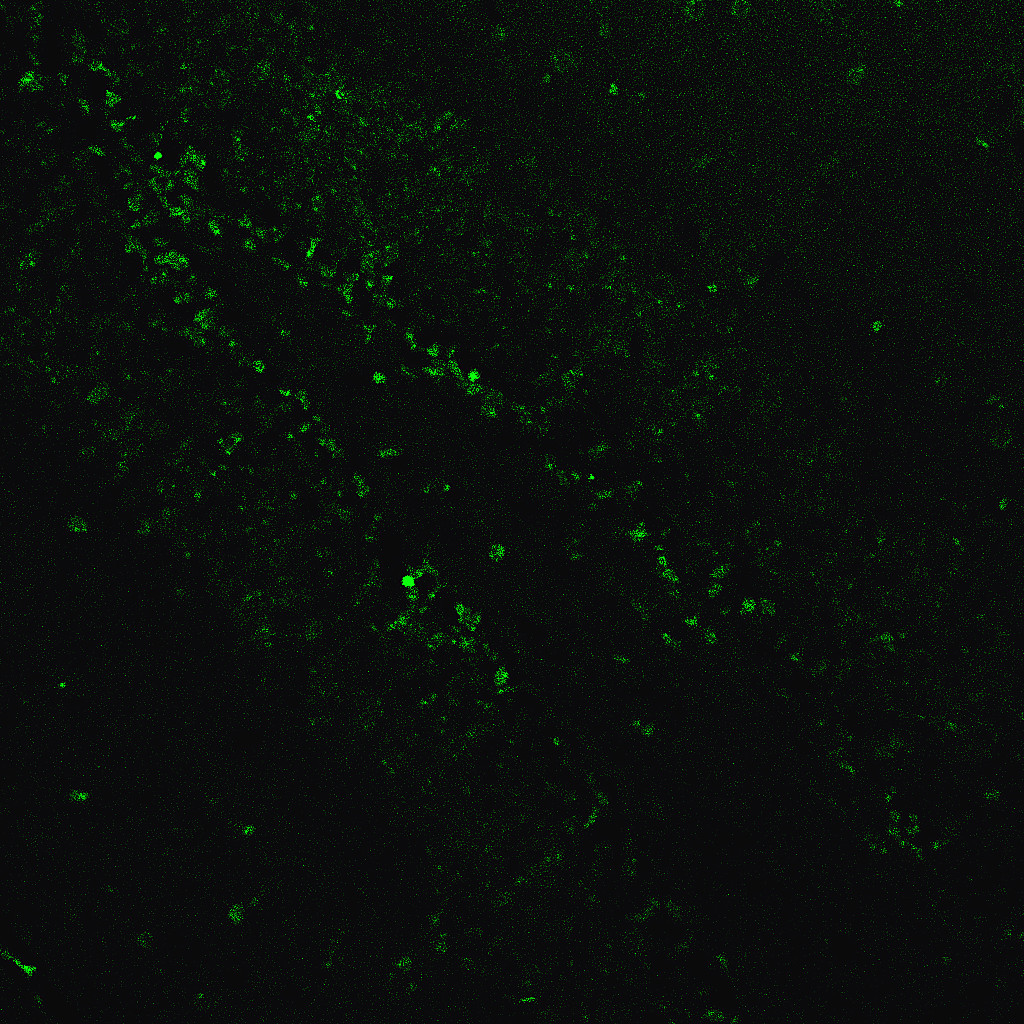 | 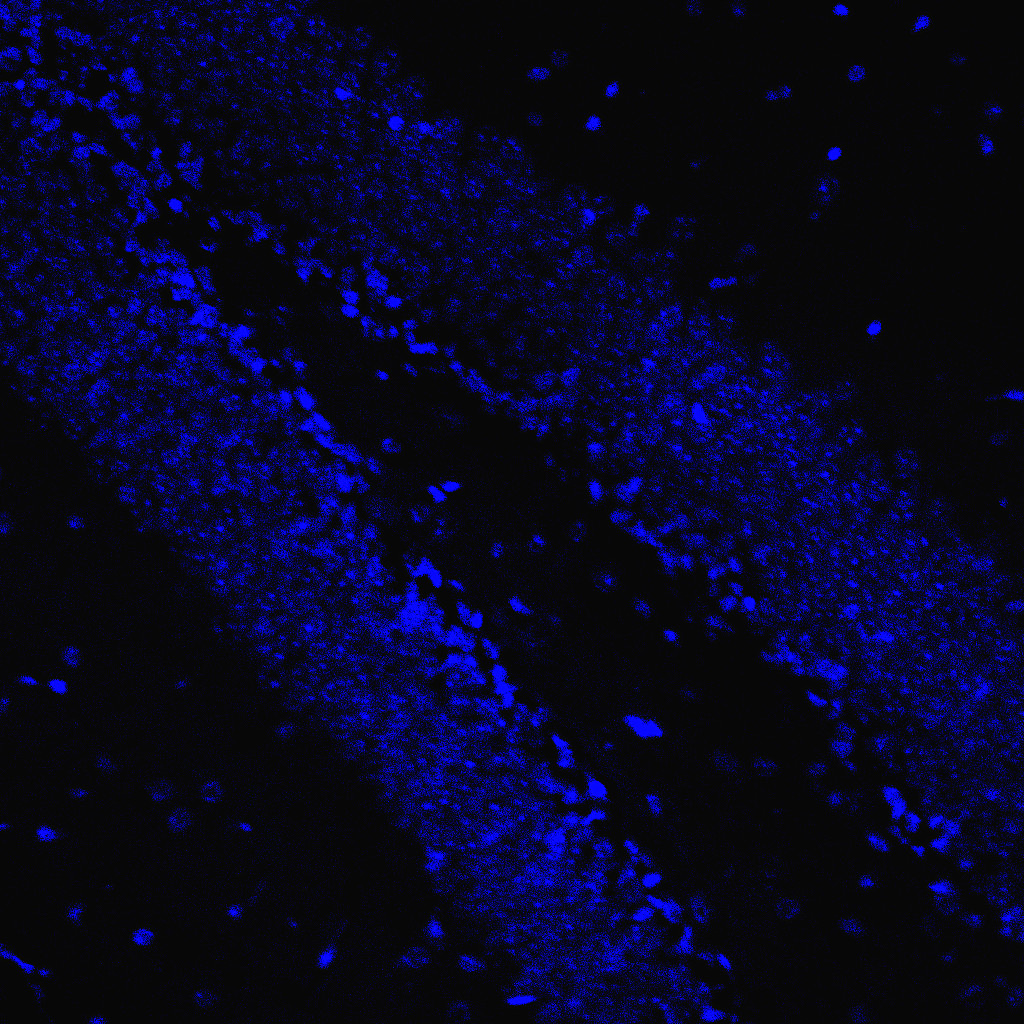 | 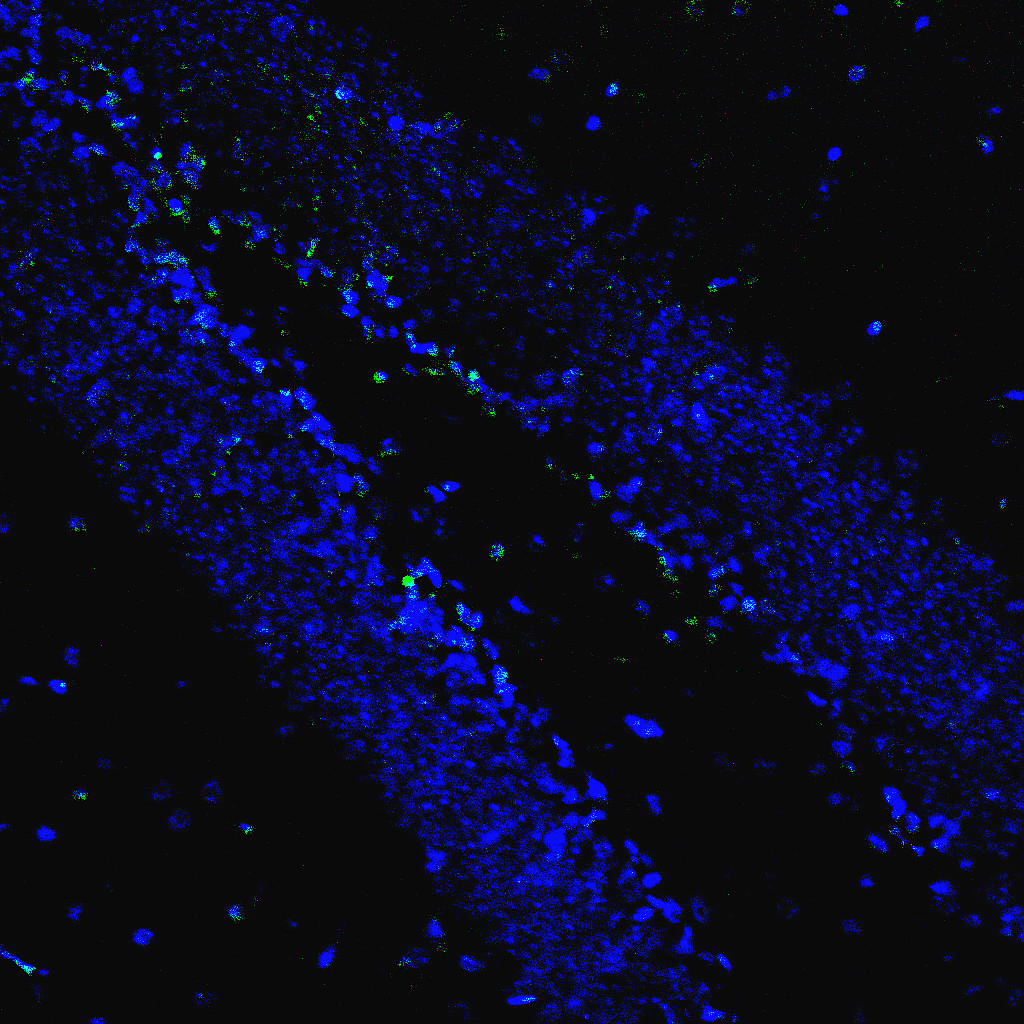 |
| **APT group** | | |
| TUNEL | DAPI | Merged |
| 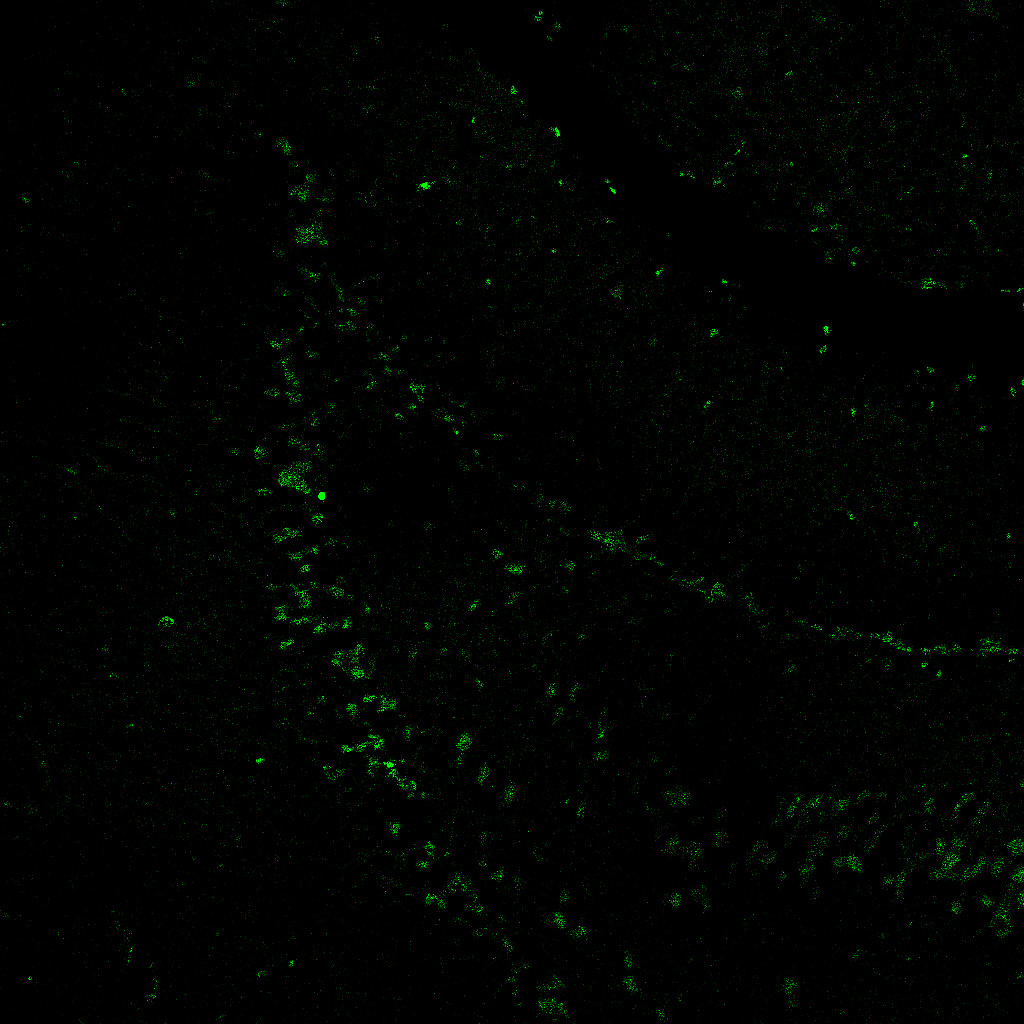 | 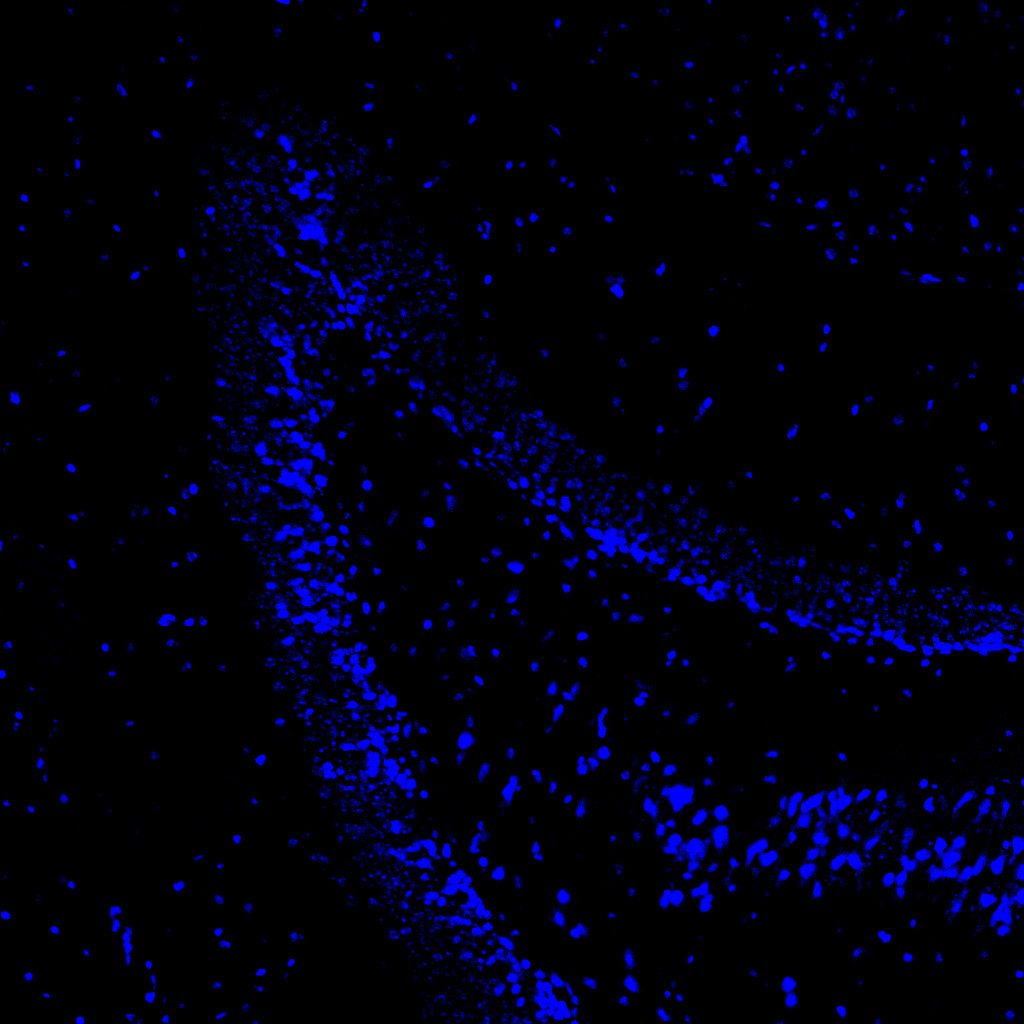 | 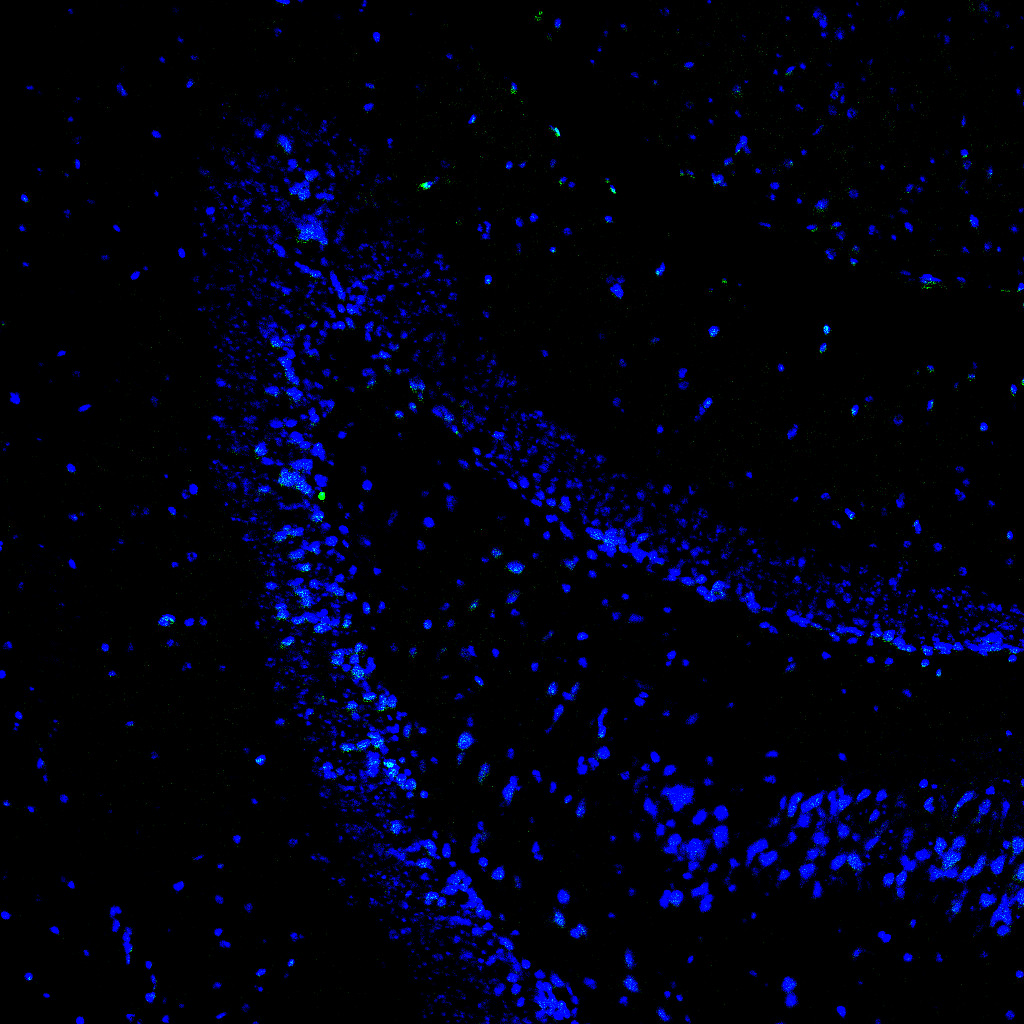 |

Supplementary Figure 4. The original image of TUNEL and DAPI staining in DG regions of the hippocampus(20×).
